# Optical pattern generator for efficient bio-data encoding in a photonic sequence comparison architecture

**DOI:** 10.1371/journal.pone.0245095

**Published:** 2021-01-15

**Authors:** Saeedeh Akbari Rokn Abadi, Negin Hashemi Dijujin, Somayyeh Koohi

**Affiliations:** Department of Computer Engineering, Sharif University of Technology, Tehran, Iran; Tianjin University, CHINA

## Abstract

In this study, optical technology is considered as SA issues' solution with the potential ability to increase the speed, overcome memory-limitation, reduce power consumption, and increase output accuracy. So we examine the effect of bio-data encoding and the creation of input images on the pattern-recognition error-rate at the output of optical Vander-lugt correlator. Moreover, we present a genetic algorithm-based coding approach, named as GAC, to minimize output noises of cross-correlating data. As a case study, we adopt the proposed coding approach within a correlation-based optical architecture for counting k-mers in a DNA string. As verified by the simulations on Salmonella whole-genome, we can improve sensitivity and speed more than 86% and 81%, respectively, compared to BLAST by using coding set generated by GAC method fed to the proposed optical correlator system. Moreover, we present a comprehensive report on the impact of 1D and 2D cross-correlation approaches, as-well-as various coding parameters on the output noise, which motivate the system designers to customize the coding sets within the optical setup.

## 1. Introduction

Sequence alignment is one of the basic bioinformatics tools for studying and analyzing biological data. Sequence aligners compare two or more genetic sequences (DNA, RNA, or protein), to discover their similarities and differences. The alignment of genetic sequences is adopted in various applications, such as DNA sequencing, DNA fingerprinting, pathogen detection, gene detection, tracing disease and cancer, tracing ancestry and evolutionary, and drug designation [1–3]. The aforementioned variety of applications alongside the ever-increasing requirement in sequencing high volume of large biological data has led to the importance of designing a fast, accurate, and scalable sequence alignment tool.

So far, various alignment tools have been developed utilizing various methods differing in terms of input sequence size, level of achieved similarity, gap and mutation treatment, type of alignment (either global or local), speed, and accuracy [4]. So far, almost all proposed alignment algorithms can be categorized into four groups [5, 1]; (1) Dynamic Programing (DP)-based algorithms (e.g. Smith–Waterman method [6]), (2) hash table-based algorithm for k-mers (e.g. PSI-BLAST [7]), (3) suffix trees-based algorithm (e.g. MUMmer [8]), and (4) cross-correlation-based algorithms (e.g. MAFFT [9], modified MAFFT [10], and Satsuma [11]).

All the above-mentioned methods meet a trade-off between efficiency (speed, memory usage, processing equipment and etc.) and accuracy to achieve an applicable and accurate method for large genome data. Implementing various algorithms using computer processors, especially in the case of massive database applications, enforces usage of a large number of robust processing cores, high CPU runtime, and expensive hardware equipment consuming vast amount of electrical energy. As the side effects of high energy consumption of electrical systems, we require strong cooling fans that, in addition to financial cost, are associated with environmental degradation.

Fortunately, solving all aforementioned issues can be achieved by introducing an emerging processing technology, i.e. optical technology, which offers two intrinsic features: 1) ultra-high speed, and 2) inherent parallel processing capabilities [12]. Moreover, due to the parallel processing and simplified implementation of many complex mathematical operations (e.g. calculating Fourier transforms of an image only by passing light through a convex lens), it can reduce the memory requirements of many algorithms. Reduced runtime, along with the ultra-low power consumption of optical processing leads to much less energy consumption in optical systems compared to their alternative electrical counterparts [13].

Specifically, recent studies have proposed optical correlators for performing biological sequence alignment [4, 14, 15]. It is worth noting that implementation of an optical correlator is not only helpful in the field of molecular biology, but also can ease data comparison within various signal processing areas, such as speech recognition, computer science [16], and image recognition (e.g. face detection [17], fingerprints detection [18], diagnosis of diseases and tumors [19], and industrial and technical applications).

To design sequence alignment architecture, two choices should be made: 1) data encoding and 2) algorithm. Although a few studies [20, 21] compare various data encoding schemes in terms of accuracy of sequence alignment, they simply adopt numerical calculation through electrical computers. Moreover, evaluating sequence similarities based on hamming distance, the aforementioned studies miss recently proposed alignment methods, taking advantages of cross-correlation [4]. Therefore, in this paper, we investigate the impact of data encoding on the accuracy of cross-correlation-based methods. Moreover, considering various encoding parameters affecting accuracy of cross-correlation, we present a novel optical code generating method. The proposed code generator, referred GAC (Genetic Algorithm based Code generator), takes advantages of genetic algorithm to generate suitable optical pattern customized for various bio-sequence analyses. As the key advantages of GAC, it is capable of producing optical patterns with different sizes in 1D or 2D formats for several numbers of letters to meet the trade-off between SNR of the cross-correlation output and the input coding efficiency Specifically, it can generate optimized codes for various bio data, such as DNA sequences (with a set of 4 letters) or protein sequence (with a set of 20 letters). In summary, key contributions of GAC method are listed as follows.

1. Generating optimal 1D and 2D encoding sets, with flexible size and parameters, targeted at the cross-correlation based pattern detectors.
2. Creating a meaningful peak value at the output of cross-correlation based pattern detectors useful for measuring the corresponding mutation rate at the input.
3. Providing 95% accuracy improvement of substring detection within the inout sequences compared to alternative methods, like BLAST, especially in the case of high mutation rates (e.g. 60%).
4. Ultra-high speed optical sequence comparison, alongside improved output accuracy, comparing to alternative methods, like BLAST.

The rest of the paper is organized as follows; at first, Section 2 reviews related works and analyzes their key advantages and drawbacks. In Section 3, basic concepts, problem statement, and our proposed solution, i.e. GAC algorithm, are discussed. Finally, in Section 4, we evaluate functionality and accuracy of GAC algorithm in terms of its runtime and resultant error rate at the output image. It is worth noting that performance appraisal is performed in two ways: simulating synthetic data, as well as, realistic benchmark data.

## 2. Related work

Generally, in order to encode bio data, various numerical representation approaches can be adopted, which are categorized as follows: 1) single-value sequence mapping (e.g. integer, 2-bit binary, and 4-bit binary), which uses a one-dimensional value to display each nucleotide, 2) multidimensional sequence mapping (e.g. VOSS, and Z-Curve), which maps each nucleotide to a two-(or more) dimensional vector, and 3) cumulative sequence mapping, who combines two aforementioned mapping strategies for each nucleotide, and provide a cumulative structure by aggregating each nucleotide value in the string using a random walk model (e.g. DNA walk, and Z-Curve) [20].

Bio data encoded by either of above schemes can be sequentially processed through mathematical operations (such as Fourier transform and wavelet transformation [21]) or goes through parallel image processing [22] by optical processors [12]. Considering the latter approach, various graphical encoding methods have been developed, each targeting one of three above categories as a means of optical image processing.

As a single-value sequence mapping, in [23], the cross-correlation technique is adopted to find the degree of similarity between two images, while it provides a simple tool for facilitating motifs exhaustive search within DNA sequences. Although this method misses a sequence alignment algorithm to exactly locate indels and mutations, the simple structure facilitates its implementation. Specifically, it converts each nucleotide to a two-dimensional image made by its alphabet symbol, and hence, output accuracy considerably depends on the resolution of optical devices. Distinguishing nucleotides' coding appearance, especially "C" and "G", by this method is so hard and has been attempted to partially solve this by substituting "X" symbol to "C" in [24]. Unfortunately, both methods proposed in [23, 24] requires large input screen for big data encoding. However, Spatial Light Modulator (SLM), as the optical pattern generator, has limited space. Hence, to feed long sequences represented by “A”, “X”, “G”, and “T” letters, they must be splitted and placed on SLM through consequence cycles.

To increase coding efficiency and reduce size of the input pattern, in [14, 4], single value nucleotide coding has been proposed. In these papers, a 4-bit binary coding and integer coding of bio data are proposed, respectively, to minimize size of the input pattern on SLM. However, the latter coding efficiency comes at the cost of reduced output accuracy in terms of peak location and their heights. So, there is a trade of between output accuracy and input size on SLM.

As a cumulative sequence mapping, in [12], two-dimensional vectors, each produced by a multi-dimensional encoding scheme, resemble each nucleotide. It is worth noting that this encoding method can exactly locate the indels with the aid of a multi-stage algorithm. As the main drawback of this method, large input patterns are produced to be fed to the input SLM. Although this coding approach suffers from various problems, such as loss of information, degeneracy, the difficulty of observing the coded curves, and difficulty of visualizing long DNA sequences, it can exactly locate the indels, while the multi-stage algorithm limit its applicability.

All aforementioned studies address wavelength domain modulation of bio-data. However, unlike electrical domain, which only enables binary encoding of bio data, optics can encode the data in four different domains, i.e. phase, amplitude, polarization, and wavelength per a spatial mode [25]. As discussed in [4, 1], choosing among four aforementioned coding domains can affect SNR of the correlation output, and hence, output accuracy. For example the study presented in [15] utilizes the wavelength spectrum, while the one in [26] uses amplitude, phase, and polarization of the signal all together to encode nucleotides. The later complexity of nucleotide encoding arises from the complex optical setup and customized processing scenarios for similar tandem nucleotides. However, effectiveness of the optical pattern generated for bio data through the cross-correlation approach is not addressed so far. So, in this work, we examine the impact of several coding methods on the output of amplitude-based cross-correlation, as one of the most commonly used similarity measurement approaches used so far.

## 3. Methodology

### 3.1 Optical implementation of cross-correlation operation

**Fig 1:**
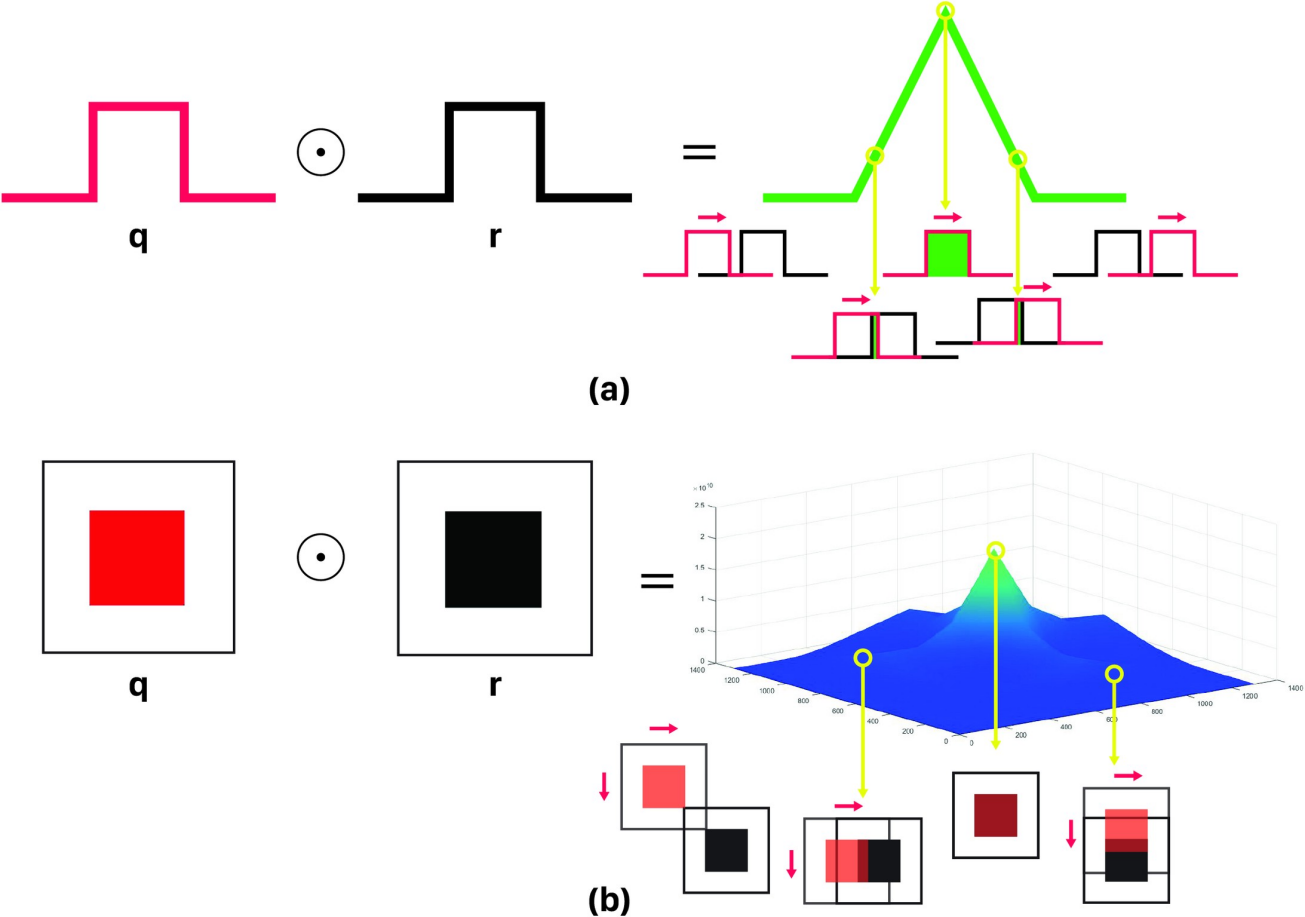
a) 1D cross-correlation, b) 2D cross-correlation.

**Fig 2:**
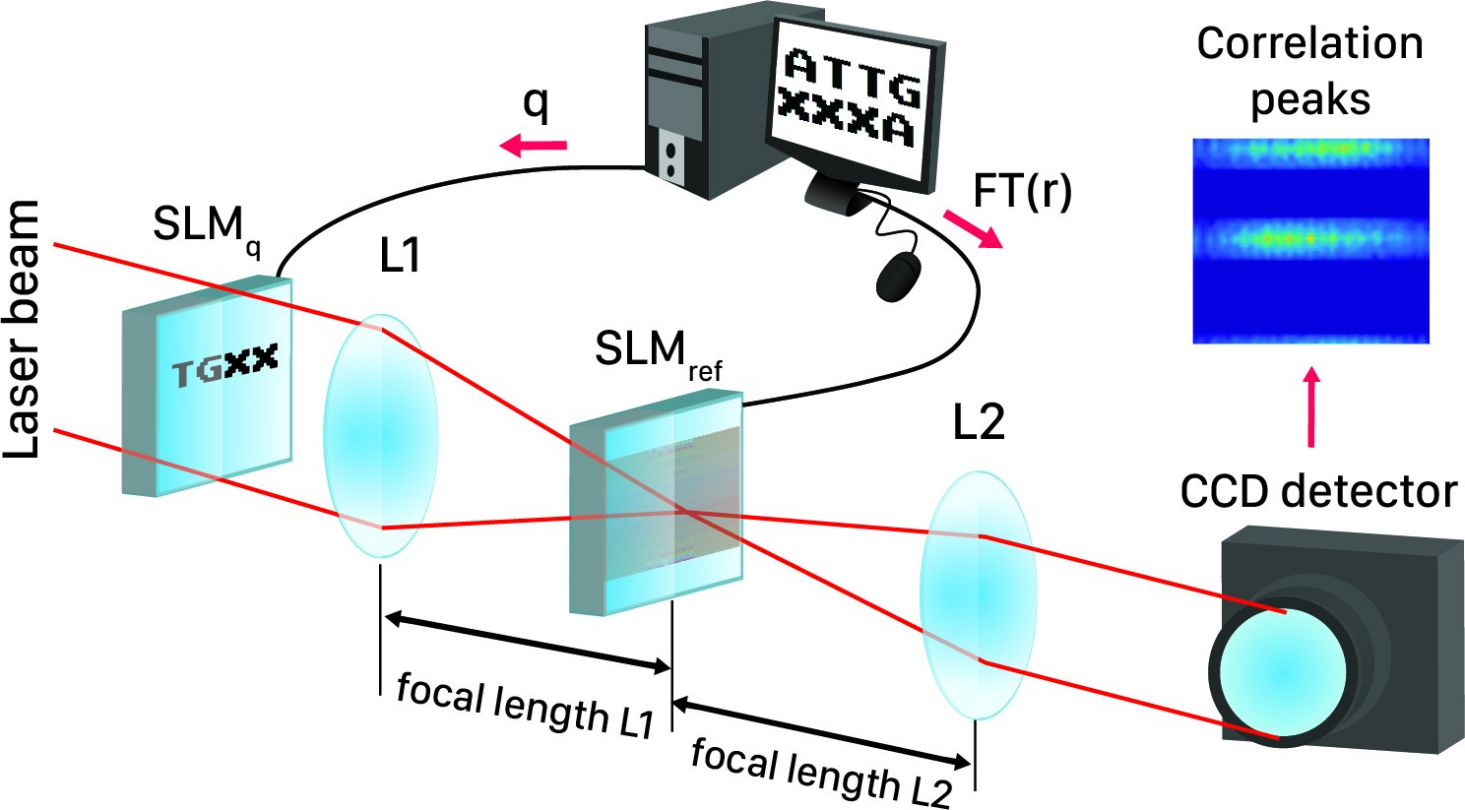
Optical setup of 2D cross-correlator for reference sequence “ATTGCCCA” and query sequence “TGCC”.

In signal processing, cross-correlation is a measure of similarity among two series of functions with relative displacement. This method is capable of applying on 1D, 2D, and 3D signals for any kind of pattern recognition application. According to Fig 1, the correlation function, either 1D or 2D, takes its maximum value when an exact replica of the query exists in the reference sequence (cross-correlation mathematics is discussed in S1 File in more details). The Cross-correlation operation in frequency domain can be implemented optically with the help of Fourier transformation property of lenses and filters. Taking advantage from parallel processing nature of optics, the Fourier transform of the whole 2D pattern would be acquired in real-time through a lens. Optical setups for both 1D and 2D correlators based on the well-known Vander lugt set-up [27] are similar and mainly lenses type may differ (1D correlator needs cylindrical lenses [28]). The general schema of these systems is shown in Fig 2. According to these figures, two SLMs, SLM_ref_ and SLM_q_, are required for putting on encoded reference and query sequences on them. As shown in Fig 2, FT of encoded reference sequence is put on SLM_ref_ and encoded query sequence is put on the SLM_q_. Given the fact that the reference sequence is kept constant in process time, without making a bottleneck during processing, FFT of reference can be computed by means of computer only once and put on the SLM. This will reduce the complexity of the optical setup.

After putting on encoded data on SLMs and installing components according to the designed setups, computations start by means of the light coming from the laser beam. Light in its path initially illuminates SLM_q_ and then passes through the lens L1. The FT of *q* (data on the SLM_q_), which is calculated by the lens L1, will be generated at its focal distance on the SLM_ref_. On the other hand, SLM_ref_ contains R, so the Dot Product of Q and R would be produced just after SLM_ref_. Finally to calculate the correlation of these two sequences, inverse FT of R.Q pattern on SLM_ref_ is computed by light passage through lens L2 and result is detected by the CCD_detector_ in its focal plane. All presented steps are shown in Fig 2 for the 2D optical correlator. In this example, encoding method of [24] is applied on input data.

### 3.2 Proposed coding scheme for optical cross-correlation

#### 3.2.1 Problem statement

As noted earlier, factors such as real-time operation and low power consumption have led us to use optical cross-correlations to solve the alignment problem. Despite determined structure of optical correlator, its various sections can still be optimized. In the following, we consider the input encoding and its effect on device performance for both types of 1D and 2D correlators. In cross-correlation devices, we deal with three types of possible noises including:

1. Overlap noise: It is the accumulated junk peaks that are produced by partial overlap of nucleotides’ coding.
2. System noise: Peaks in output may vary from their expected length by a positive error value, potentially leading to large accumulated errors in output.
3. Neighbor noise: It shows up when it is not possible to distinguish each peak in its exact location on SLM due to neighbors with nearly equal length.

**Fig 3:**
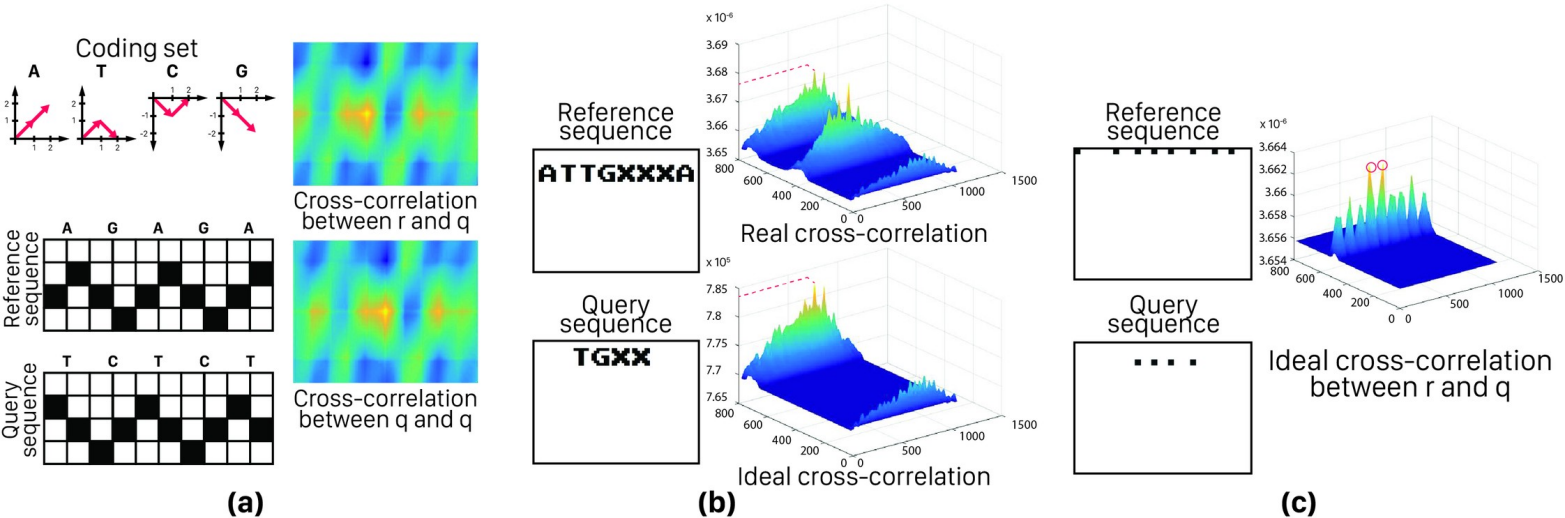
Three types of output noises; a) Overlap noise: code overlapping as the result of DV-Curve encoding method results in output peaks which mislead to sequence matching, b) System noise: output peak value decreases when realistic condition is simulating, c) Neighbor noise: adjacent peaks (as the result of either valid peaks or high-altitude noises) avoids proper indel locating.

In Fig 3 some example of these three noise types are shown. According to this figure, some facts regarding the encoding effect on the quality of results become clear. For example, 2D codes, same as Fig 3B, by providing distance between successive informative peaks can control neighbor noise, but they also cause partial overlaps, leading to overlap noise. So, encoding method may have some effect on the performance of the cross-correlator.

In general, according to Fig 3 examples, the more differentiation among codes make the output noise lesser. The exact and mathematical expression of this problem can be found in Problem 1. It should be noted, however, that this problem is more evident in 2D encoding since in both dimensions the codes are overlapping. In 1D cross-correlation, finding an answer to design codes’ pattern is similar. In this way, problem 1 is given for 2D encoding methods, for the more general structure of the problem. It should be noted that if the SLM width is less than the length of the encoded sequence, the sequence is divided into subsequences and these parts are placed under each other. Therefore, in the 2D mode, in addition to the horizontal overlaps that are created naturally by staying on alphabets side by side in the sequence, vertical overlaps also arise from putting on subsequences underneath each other.

**Fig 4:**
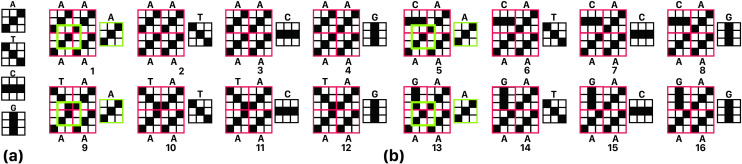
a) Example of coding patterns, b) 16 sample states of all possible 1024 states of problem 1 for C_3, 4_, assuming i = 2 and j = 2, where C_3, 4_ is a coding set containing 4 characters that each of them is a 3 × 3 matrix, i and are width and height, respectively, of a grid of codes that contains all combination of C_3, 4_. So, multiplying 4^4^ possible states, required for creating this grid, by 4 states for each code leads to 4^5^ or 1024 states. Exact matched pattern of single codes and multiple codes overlaps are shown with a green stroke rectangle.

*Problem 1*. Assume that *C*_*d*,*K*_ is a set of *K* codes with size *d × d*. According to Eq (1), this set is optimized for Overlap noise, if in addition to the maximum two-by-two differentiation, for all pairs, the highest overlap of all possible placement of alphabets in *i × j* rectangle, with each *i– 1 × j– 1* rectangle is minimized; i,j∈N−{1}. Fig 4 illustrates some states of an example of this issue for C_3_, _4_, i = 3 and j = 2.

$$\begin{array}{l} \forall i,j\epsilon N-\{1\},1\leq m,n\leq |C_{d,K}|,c_{m}\epsilon C_{d,K}:\\ \;M_{i\times j}={\left[c_{m}\right]}_{i\times j}\\ \;N_{i-1\times j-1}={\left[c_{n}\right]}_{i-1\times j-1}\\ \;X_{2i-1\times 2j-1}=M_{i\times j}*N_{i-1\times j-1}\\ \;optimal\;C_{d,K}↔minimize(x_{s,r}),\\ \;1\leq s\leq 2i-1,1\leq r\leq 2j-1,\\ \;s,r\neq \frac{hd}{2}and\;are\;odd \end{array}$$ (1)

* is cross-correlation operand

Problem 1 can be reduced to a simpler structure with less number of states as in problem 2.

*Problem 2*. Similar to Eq (2), problem 1 is true if and only if problem 1 holds for i = 2 and j = 2.

$$\begin{array}{l} \forall i,j\epsilon \{2\},1\leq m,n\leq |C_{d,K}|,c_{m}\epsilon C_{d,K}:\\ \;M_{i\times j}={\left[c_{m}\right]}_{i\times j}\\ \;N_{i-1\times j-1}={\left[c_{n}\right]}_{i-1\times j-1}\\ \;X_{2i-1\times 2j-1}=M_{i\times j}*N_{i-1\times j-1}\\ \;optimal\;C_{d,K}↔minimize(x_{s,r}),\\ \;1\leq s\leq 2i-1,1\leq r\leq 2j-1,\\ \;s,r\neq \frac{hd}{2}and\;are\;odd \end{array}$$ (2)

* is cross-correlation operand

**Fig 5:**
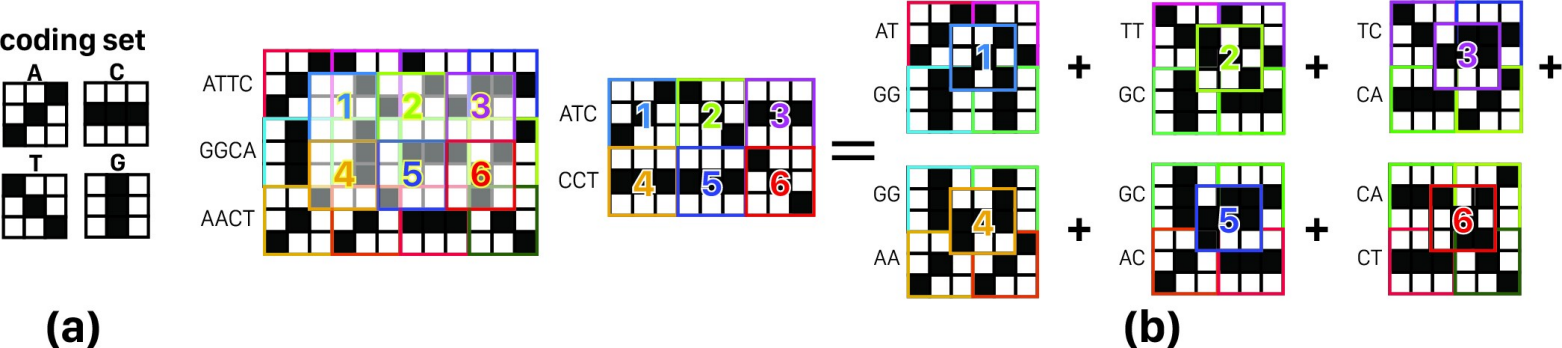
a) Example of coding set, b) problem 1 with i = 3, j = 2, and K = 3 for one step of cross-correlation; if in the left side, the sum of inner product of two grids for the shown spatial relative place of them will be calculated, its result (= 5) was equal to the sum of inner product of right side elements (right side elements are sub-grid of left side grids (= 5).

*Proof*. According to the definition of the cross-correlation in (S1a in S1 File) and the linearity of integral, cross-correlation has the ability to decompose into smaller sections to calculate a cross-correlation result matrix. On the other hand, as demonstrated as an Example in Fig 5, grids with dimensions i > 2 and j > 2 can be divided into 2 × 2 grids. The sum of the multiplication of the larger grids can be obtained by summing multiplication of sub-grids.

We define *c-grid* a 2 × 2 array of codes with a structure like [[c_1_, c_2_], [c_3_, c_4_]] where *c*_*i*_ is in {0, 1}^(d × d)^ to represent an encoded nucleotide, and *i* is in {1, 2, 3, 4}, as shown in Fig 5A. So, with the assumption that the maximum sum of multiplication (SOM) value of each *c*_*i*_ with 2 × 2 grids [[c_k_, c_l_], [c_m_, c_n_]] are minimized, their summation is also the lowest possible value. Because if this were not true and there existed an SOM lower than the obtained value, it meant that at least one of the sub-grids' SOM values could be still minimized and this is in contradiction with the initial assumption.

In next sections, we will propose parameters for 2D codes which put a certain upper bound on ratio of partially overlapping peaks to informative full peaks in order to control Overlap noise. These parameters will also create a margin for System noise and neighbor noise to help extracting every information from informative peaks.

#### 3.2.2 GAC (Genetic Algorithm based Code generator) coding algorithm

**Fig 6:**
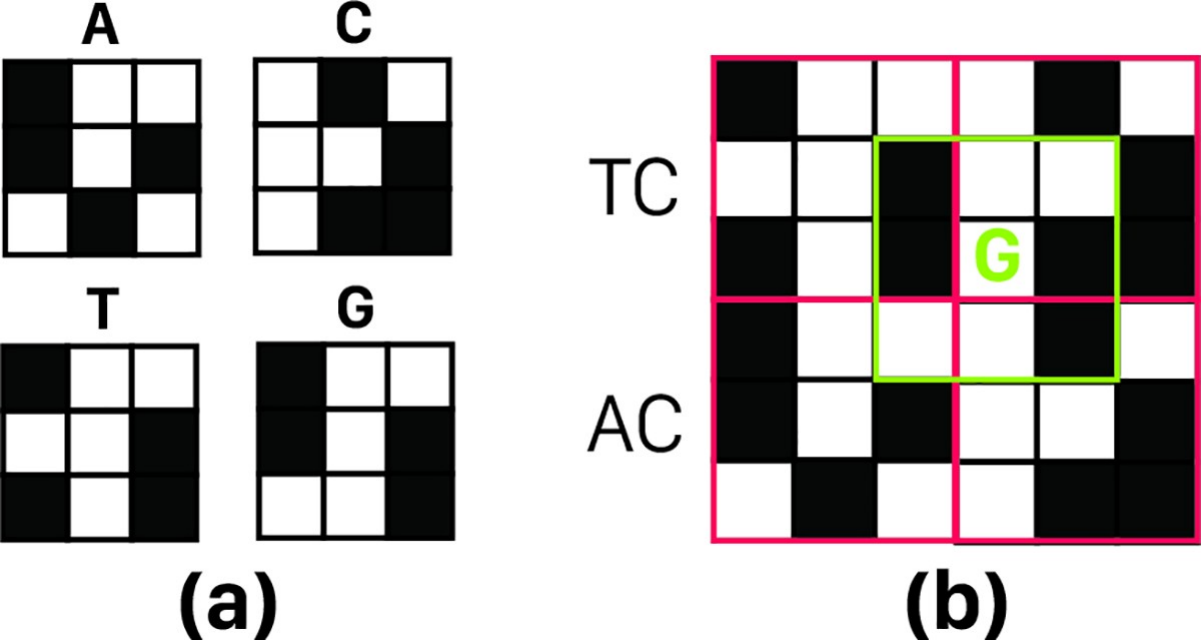
(a) Set of four codes with N = 4, d = 3. (b) a c-grid (2 × 2 grid) with an overlap noise of length four, so if E = 1 will be chosen. This coding set is unacceptable (because size of peak in this example is 4 too and 4—E (= 1) is the maximum acceptable noise); overlap noise location is marked with a green border.

We assume that each c_i_ code has N ones; so, by sliding a single nucleotide over a c-grid, maximum length of resulted peak would be N that we call it "full peak" or "informative peak". Accurate full peak detection requires significant difference between expected length of full peak, and non-full peaks as a safety margin for System noise. We represent this difference by E. Acceptable set of codes for nucleotides should produce no peak longer than N–E by sliding any nucleotide on anyone of 256 c-grids obtained from that set (where each c_i_ is one of the proposed d × d arrays for A, C, T, or G). Such a peak is considered as an invalid peak. An example of an invalid peak is shown in Fig 6 for Set of four codes with N = 4, d = 3 and acceptance threshold E = 1. Obviously, there would be a full peak when sliding nucleotide matches itself on c-grid, which is not only valid, but also our main target.

Given problem 2 and defined parameters d, N and E, in order to evaluate different answers and ultimately obtaining the appropriate answer, we need to aggregate the parameters in a metric; this metric is indeed a cost-function which could be considered as the number of invalid peaks for a specific set of four codes and a pre-determined value of E. The minimum value of this cost-function would yield the best set of four-codes.

Summarizing above discussion, our problem can be considered as an optimization problem, and thus could be solved using the well-known optimization algorithms. However, while there exist various optimization algorithms, each of them is compatible with a specific type of problem within a field application [29, 30], and they cannot be necessarily interchanged for solving another problem. It should be noted that the choice is made after a comprehensive study of machine learning (ML) methods, to conclude that our problem cannot be interpreted as a solvable ML's problem or other optimization algorithms. In this paper, we propose a code generation method based on genetic algorithm, as a compatible optimization algorithm with our choice of problem, which is finding a coding set with least value of the defined cost-function for the given inputs values of N, E, and d. Choosing inputs for different applications and devices may vary according to their requirements. This approach is functional even for large values of d, and Ns; or with numerous types of biological alphabets like amino acids in protein sequence. Although it is time-consuming, it needs to be executed only once before main application; thus, it is useful.

Canonical form of Genetic Algorithm needs an initial population of encoded individuals. In every evolution cycle, two individuals are selected from the generation to be the parents, based on a specific criterion. After applying crossover function on parents, a mutation occurs on two new individuals with a particular probability, and they may join the next generation. The cycle continues to repeat until termination condition has not been met [31]. In this work, we alter some of these steps and elements to apply constraints, and prevent population from deterioration [32].

1. *Population*, *and individuals*. Each individual (I) represents unique location of ones in correspondent set of four codes; thus I is in {1,…, d^2^}^{4N × 1}^, and has four chunks: I[1:N], I[N+1:2N], I[2N+1:3N], and I[3N+1:4N]. Initial population is developed from a random initial individual by repeated mutations. In order to obtain possibly better population, initial individual can be specifically highly fitted.
2. *Mutation*. Mutation should preserve the aforementioned features for mutating individuals. So, it has the following steps:
  - Choosing from a random chunk,
  - Picking a random entry in the chunk,
  - Changing the selected entry to a number in {1,…, d^2^} which is not already in the chunk.

This approach ensures that each chunk will have N unique entries representing locations inside a d × d code.

3 *Fitness evaluation*. Objective function is defined to be -1 × cost-function; thus, individuals with less value of cost-function have higher fitness.
4 *Selection*, *and crossover*. Selection occurs by choosing two individuals with highest fitness from current generation. Crossover only happens between chunks to maintain critical characteristics described for individuals. Offspring individuals join the next generation only if both have higher fitness compared to worst member of current generation; otherwise parents remain unaffected, and crossover does not change generation in current cycle [31].

So, after initiating population, evolution begins, and continues until an individual with a fitness of zero is produced. In this way, we define a new term called as “zero-score coding” as follows, to be used in cost function calculation through the proposed algorithm.

Definition 1: Zero-score coding: Coding set with the peak noise value of N—E.

The overall procedure is summarized in Algorithm 1.

## 4. Simulation results and analysis

This section evaluates GAC method from three different points of view; a) its runtime, b) effectiveness of coding sets genereted by GAC method on cross-correlation output noise, and finally, c) accuracy of DNA string matching by adopting GAC method for realistic bio data encoding. Of course, in order to evaluate our work, we also conducted more experiments, such as checking the accuracy in counting patterns and optic effect on output by doing optical simulation with zemax, which is discussed in Section ‎4.3.5.

### 4.1 Runtime analysis

**Fig 7:**
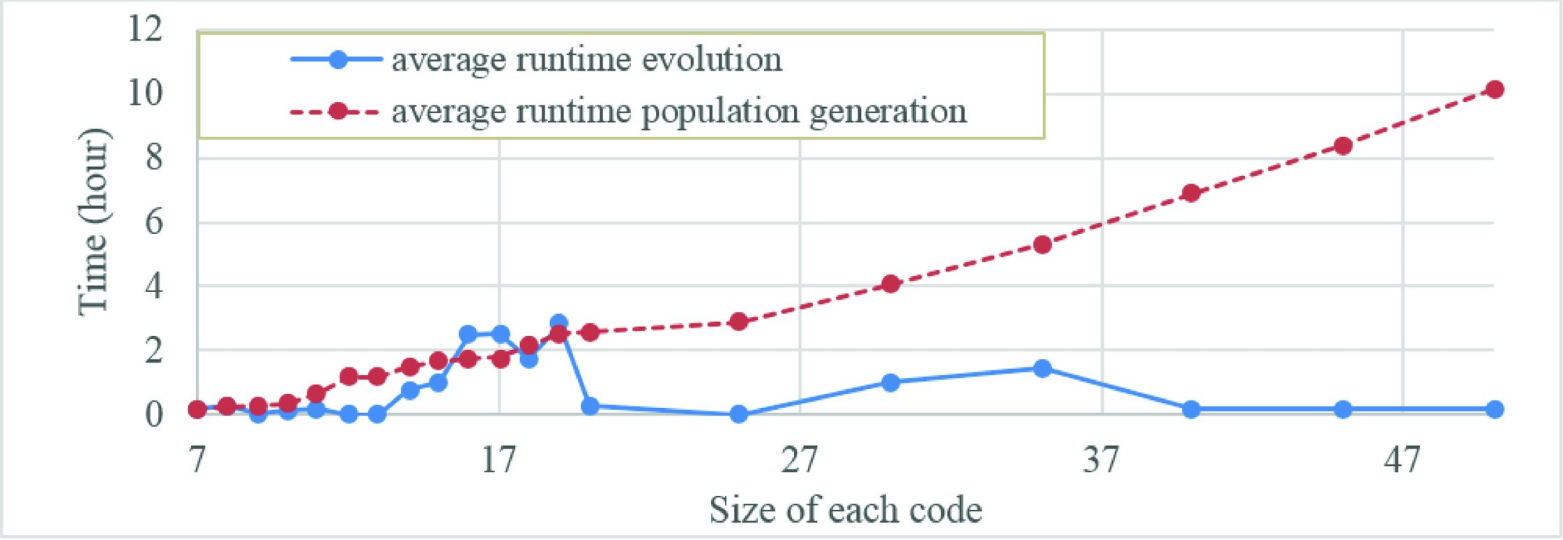
Evolution and generation runtimes during search for various sizes of zero-scored 1D code.

**Fig 8:**
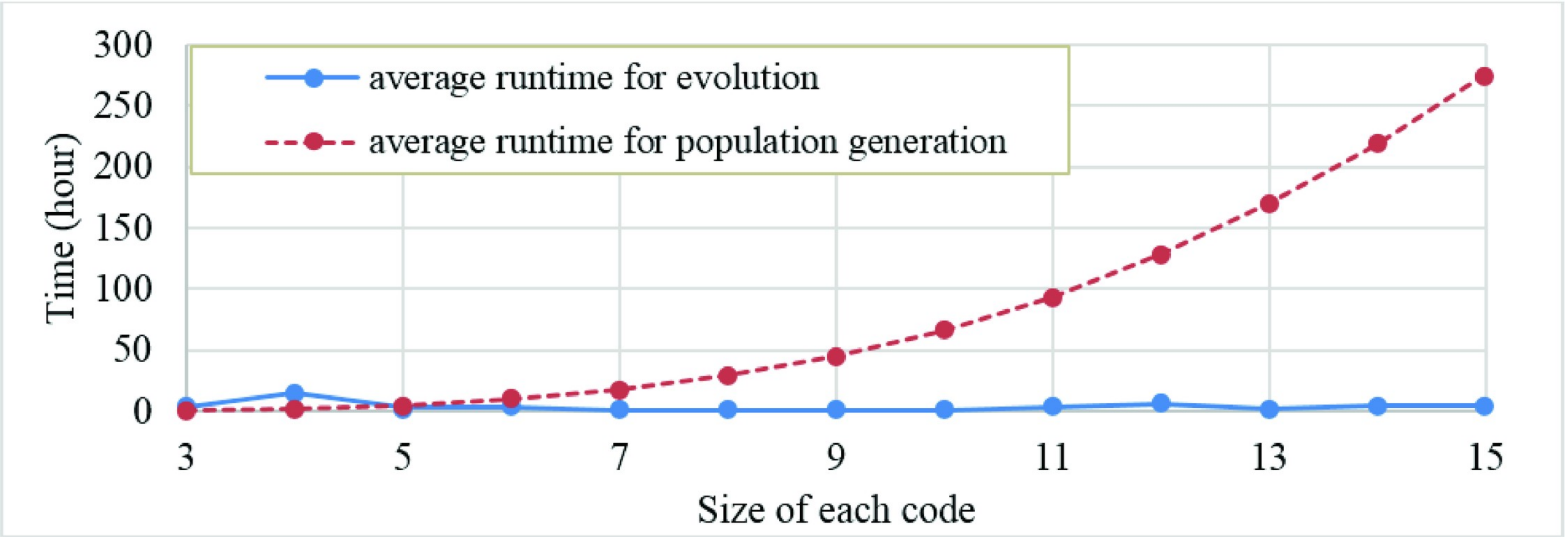
Evolution and generation runtimes during search for various sizes of zero-scored 2D code.

As a usual trend for reporting runtime of the genetic algorithms, we calculate and report its average runtime for various input data sets. Runtime variation for both code sizes, i.e. 1D and 2D, is depicted in Figs 7 and 8, respectively. According to these figures, larger code size leads to slower population generation, but speedup the evolution. Accordingly, generation phase considerably impacts time complexity, especially as code size increases. Figs 7 and 8 show that the runtime of genetic algorithm increases linearly and quadratically with the code size in the case of 1D and 2D output patterns, respectively.

### 4.2 GAC algorithm parameters analysis

In this section, we addresses how different choices of code features; i.e. d, N, and E, affect output noise of the cross-correlation. Details of these evaluations are reported in a Section entitled as "GAC algorithm parameters analysis” of the S1 File.

Finally, efficient ratio depends on devices and their applications; thus, choice of suitable parameters for the algorithm depends on existing tradeoffs between length of sequences and required ratio for accurate analysis of device output. For example, one criterion for choosing E is system noise amount in device. Considering ε as the least upper bound for system noise in the longest possible peak on output surface, so 2ε becomes a lower bound for E; because least difference between two encoded strings, i.e. a single mutation, should be confidently detectable. A lower bound on E restricts choice of size, and target ratio. Of course, for d > 10 and small values of N, the random mode of generating codes will often receive an acceptable score. It might seem that there is no need to use the GAC method, but the point is, in fact, we pay the area-cost of occupying more space on SLM by larger coding instead of the time-cost of the GAC method. However, by doing this, we increase execution time of the cross-correlation due to the more number of data switching on SLM in the case of long sequences, and so, randomized coding sets do not necessarily meet the time limitations.

Concluding the section, Table 1 summarizes the impact of each parameter, i.e. relative threshold, N, and d, on different metrics evaluating a proper coding set. It must be noted that some metrics for parameter analyzing are defined in S1 File that one of them that is mentioned here is Relative threshold as defined in Definition 2.

**Table 1:** Effectiveness of optimizing triple parameters (i.e. relative threshold, E, and N) and coding metrics.

Metrics	Relative threshold	N	d
**Overlap noise**	Inverse	Small N: Inverse, Large N: direct	Inverse
**Neighbor noise**	Inverse	Small N: Inverse, Large N: direct	Inverse
**Number of zero-score coding sets**	Inverse	Small N: direct, Large N: Inverse	Direct
**Population time**	Direct	Small N: Inverse, Large N: direct	Direct
**Evolution time**	Direct	Small N: Inverse d, Large N: direct	Inverse

Definition 2—Relative threshold: Since the absolute value of peak acceptance threshold (E) depends on the size and number of 1 bits of each coding set, the relative threshold is defined as the ratio of E to N to eliminate this dependency.

By defining relative threshold, all codings with any values of triple d, N, and E become comparable.

### 4.3 Evaluation and comparison

To clarify the impact of optimized optical coding set on the accuracy of pattern recognition and alignment process of biological data, in this section, we evaluate GAC method against alternative coding methods. Specifically, we introduce evaluation metrics to compare accuracy of DNA string matching utilizing the coding sets generated by GAC method against those of alternative approaches.

#### 4.3.1 Overall view of coding methods’ features

Table 2 summarizes coding features of the aforementioned methods. According to this table, a suitable coding method is the one that: 1) uses SLM's 2D space efficiently to fill it with more data, 2) makes size, shape, and value of coding set flexible according to the limitations of the optical system(like detector resolution), 3) generates customized alphabets set with flexible size for various input data (e.g. DNA with 4 alphabets, RNA with 4 alphabets, and protein with 20 alphabets), 4) minimizes overlap noise among optical codes for reducing cross-correlation overall noise, and finally, 5) while it produce sharp peaks at the output of cross-correlation, it results in more useful data at the output image, such as peak lengths, meaningful for more post-processing.

**Table 2:** Related methods’ features summary.

Coding Methods	Coding sets	Features				
		SLM usage	adaptable with optical setup limitations*	scalability of the coding set	Overlap noise	Meaningful Peak
[23, 24]	Alphabet symbols	2D	No	Yes*	Yes	No
[33]	Integers in the range of 0–255	2D	No	Yes*	No	No
[14]	1×4 cell arrays with a single entry equal to one	2D	No	Yes*	Yes	Yes**
[12]	Double DV-curves	1D	No	No*	Yes	Yes

* While these papers do not discuss scalabilty of their coding set, it can be elicited they might offer this feature

** Depending on the scaling method adopted, this coding approach might produce meaningful peaks at the output.

To provide various desirable features of an appropriate coding method, as listed in Table 2, we propose GAC, Genetic Algorithm-based Code generator, as a code generator tool based on genetic algorithm. GAC can generate 1D and 2D (square-shape) coding sets with arbitrary symbol size (features 1 and 2) and arbitrary number of valuable bits (feature 2 and 5) for various ranges of alphabet set size (feature 3). Moreover, GAC cost function targets minimizing overlap noise among various coding symbols (feature 4).

As follows, we illustrate efficiency of GAC method to reduce overlap noise and produce meaningful peak at the output of a pattern recognition system built upon either electrical or optical cross-correlation approach. For this purpose, we evaluate two of our proposed coding sets, generated by GAC method, utilizing evaluation benchmarks of [33], and compare accuracy of our results against that of [33] and BLAST method.

#### 4.3.2 Preliminaries of evaluation

**Fig 9:**
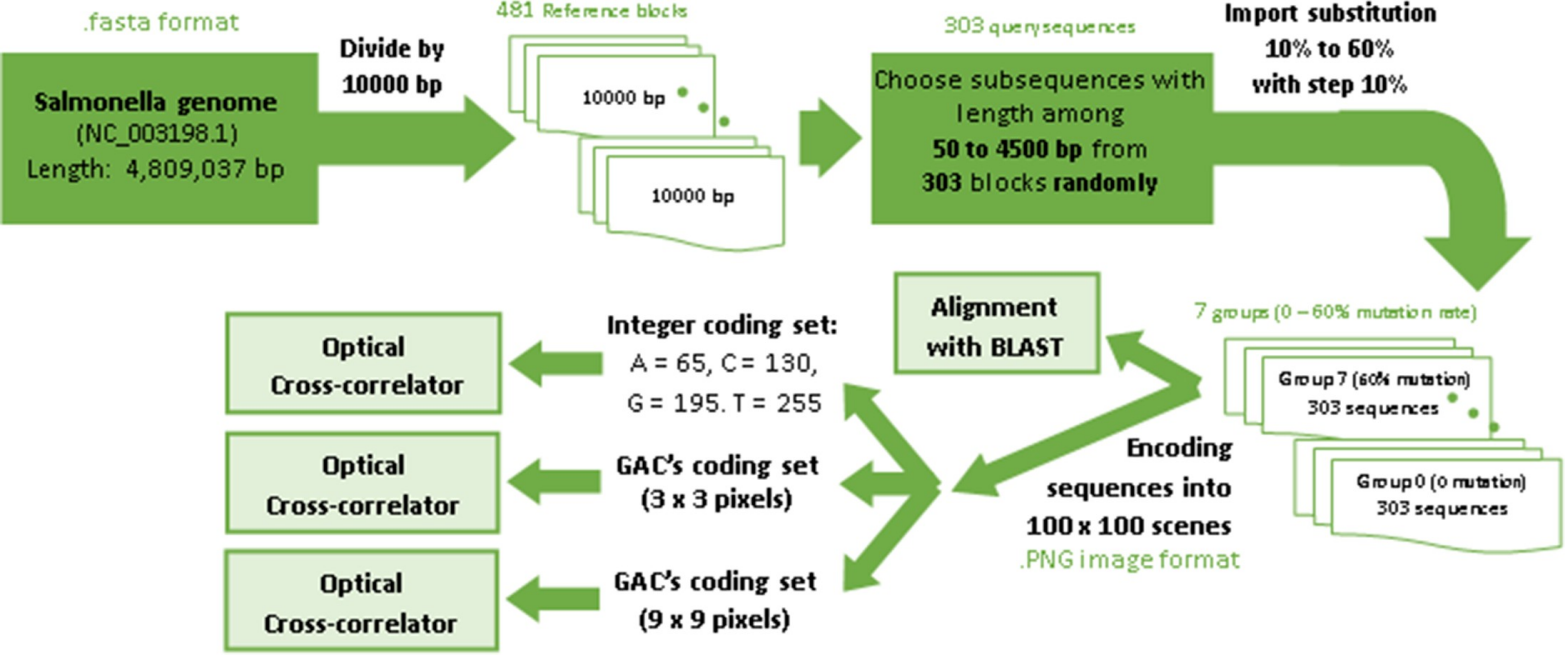
Evalution steps.

For efficiency comparison, we encode the whole-genome of Salmonella with access code NC_003198.1 in NCBI database. Its genome, containing 4,809,037 bp, is divided into 481 scenes each with a size of 100 × 100 bp^2^. However, it is worth noting that in [33], only a single section of one million bp is used, leading to smaller searching space compared to ours. As the next step, 303 randomly chosen subsequences with the length randomly chosen in the range of 50 bp to 4500 bp are selected from this genome. Nucleotide substitution with the rate up to 60% (with step-size of 10%) manipulates these 303 sequences; so 2121 sequences are created with randomly located substitutions. Finally, cross-correlation is used as a pattern recognition tool to compare all 481 scenes with all 2121 generated sequences as queries. Fig 9 briefly show all step of evaluation GAC method.

For encoding Salmonella genome, we choose two zero score coding sets generated by GAC; one with the size of 3 × 3 with three bits equal to 1 and E equal to 0, and another one with the size of 9 × 9 with 32 bits equal to 1 and E equal to 11.

To evaluate accuracy of the alignment methods proposed in [33], four metrics are defined as follows; 1) Sensitivity (Se) to measure correct diagnosis of queries’ existence, 2) Specificity (Sp) to measure correct diagnosis of queries’ absent, 3) Exactitude (Ex) to measure correct diagnosis (both present and absent), and 4) Error (Er) to measure incorrect diagnosis (both present and absent). Their formulas as represented by Eqs (3) to (6) are utilized for reporting our results.

$$Se=\frac{TP}{TP+FN}$$ (3)

$$Sp=\frac{TN}{TN+FP}$$ (4)

$$Ex=\frac{TP+TN}{n}$$ (5)

$$Er=\frac{FP+FN}{n}$$ (6)

Where TP (True Positive) represents the number of existing queries identified correctly; TN (True Negative) is the number absent queries identified correctly; FP (False Positive) represents the number of absent queries mistakenly identified as existing queries; FN (False Negative) is the number of existing queries mistakenly identified as absent queries; and finally, n is the size of search-space.

To evaluate accuracy of various detection methods based on above aforementioned metrics, some test scenarios, including two types of correct (positive) or incorrect (negative) answers, are designed. It should be noted that since the true answer for each test scenario is pre-known, going through various detection metrics, we can precisely specify which metric leads to accurate pattern detection at the output. Specifically, the "sensitivity" metric defines the percentage of positive answers correctly diagnosed as positive; thus, the most accurate detection method should result in sensitivity value of 100%.

Moreover, in addition to full detection of positive cases, an accurate detection method must be able to specify negative cases, and label them as negative. This capability is quantized by "Specificity" metric. This metric is defined as the percentage of negative answers correctly diagnosed as negative; thus, the maximum value of Specificity equals 100%. Considering above discussion, we can conclude that measurement of both metrics, i.e. sensitivity and specificity, is necessary for precise evaluation of a detection method. Finally, "Exactitude" metric provides an overview of output accuracy and accurate diagnosis percentage of the method, while "Error" metric measures the percentage of wrong answers produced by the detection method. Therefore, 100% is the best value of "Exactitude" metric, while 0% is the best "Error" value. As follows, we discuss our comparative simulation study.

#### 4.3.3 Quadruple metrics

As discussed before, 303 queries are manipulated with various substitutions rate in the range of 0 to 60%, while pattern recognition is performed by cross-correlating each category with the reference sequence. We can compute the aforementioned Quadruple evaluating metrics for each substitution rate separately, as listed in Table 3.

**Table 3:** Quadruple evaluating metrics for different mutation rates (%)– 3 × 3 coding set.

noise	0	10%	20%	30%	40%	50%	60%
Se	100	100	100	100	99.34	97.69	86.47
Sp	100	100	100	100	99.998	99.995	99.972
Ex	100	100	100	100	99.997	99.990	99.944
Er	0	0	0	0	0.0027	0.0096	0.0563

According to these results, all metrics verify ideal pattern recognition up to mutation rate of 40%, beyond which error value (represented as er) slightly increases, while it is still negligible. Specifically, although the error rate increases with mutation rate increment, its growth rate not noticeable.

Analyzing error cases, we can conclude that error rate increases for short queries. Specifically, increasing the mutation rate increases the probability of erroneously locating the true peak within the short queries. On the other hand, it should be noted that as the mutation rate increases, the maximum query length that may not be identified correctly also increases. According to this fact, we can conclude that a coding set with higher signal to noise ratio (as represented by "E" in the GAC method) improves the maximum length of a query correctly identified for a specific mutation rate, noting that the maximum length is decreased for larger mutation rate. In this manner, we verify the aforementioned conclusion, and rerun the simulation scenarios for searching cases and encoding queries with a larger coding set (I.e. zero-scored coding set with size 9 × 9, 32 bits one and E equals to 11). The evaluation results are depicted in Table 4. As expected, using a coding set with higher E, and so higher signal to noise ratio, leads to more accurate peak value detection, and hence less cross correlation error. According to this table, larger coding sets leads to negligible error rate assuming mutation rate up to 50%.

**Table 4:** Quadruple metrics under different mutation rates (%)– 9 × 9 coding set.

noise	0	10%	20%	30%	40%	50%	60%
Se	100	100	100	100	100	99.340	95.380
Sp	100	100	100	100	100	99.999	99.990
Ex	100	100	100	100	100	99.997	99.981
Er	0	0	0	0	0	0.0027	0.0192

### 4.4 Comparative studies

In this section, we compare the accuracy and speed of the proposed optical cross-correlation system fed by the coding sets generated by GAC method, optical cross-correlation that uses [33] integer coding set, and finally, BLAST (2.9.0+.BLAST version). All simulations are performed within MATLAB 2016a simulation environment on a system with 2.20 GHz Intel(R) Core(TM) i7-2670QM CPU.

#### 4.4.1 Accuracy

Although the aforementioned evaluating metrics behave similarly in the case of integer coding [33], the proposed integer coding achieves high processing speed at the cost of reduced output sensitivity; specifically, in the case of 60% mutation rate, the output sensitivity reduces to zero. Table 5 summarizes cross-correlation accuracy, in terms of previously defined evaluation metrics, under various mutation ranges.

**Table 5:** Quadruple metrics under different mutation rates for integer coding [33] (%).

noise	0	10%	20%	30%	40%	50%	60%
Se	99.01	98.35	97.69	95.05	88.12	42.52	0
Sp	98.99	99.98	99.98	99.98	99.98	99.99	99.98
Ex	99.98	99.96	99.96	99.93	99.86	99.42	98.98
Er	0.02	0.04	0.04	0.04	0.14	0.58	1.02

**Fig 10:**
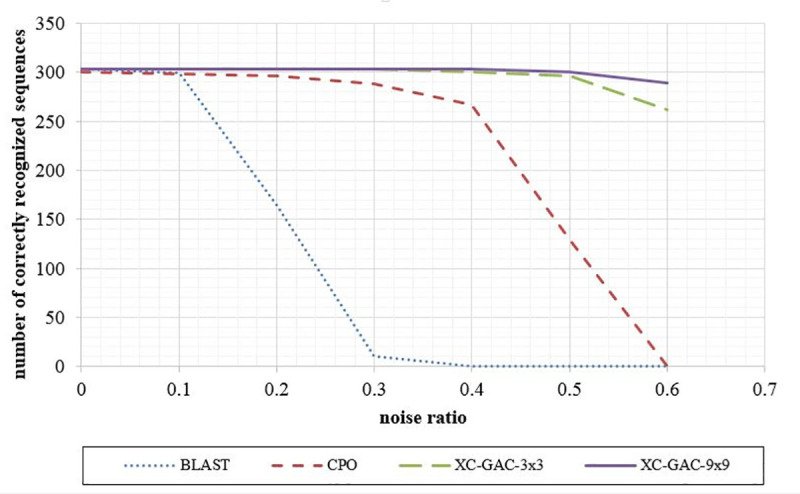
Snetivity and mutation rates for three method; BLAST, cross-correlator based on integer coding set (CPO), and cross-correlator based on GAC coding set (XC-GAC).

As follows, we compare the accuracy of the optical cross-correlation system assuming four different input pattern generation approaches: a) GAC approach with with 3 × 3 code sets, b) GAC approach with 9 × 9 coding sets, c) integer coding set, and d) BLAST method to clarify GAC advantages. According to the small number of true positive cases, compared to the search space size, sensitivity is the most meaningful metric to evaluate, and hence, we choose it for comparing various coding approaches. Output sensitivity of an optical correlator with integer coding set vs. BLAST is reported in [33]. On the other hand, as shown in Table 6 and Fig 10, sensitivity values of an optical correlator fed by coding sets generated by GAC method slightly change with the mutation rate, while the integer coding method, as well as the BLAST coding approach lead to considerably reduced sensitivity with mutation rate growth. Finally, as discussed before, 9 × 9 coding sets also outperforms 3 × 3 ones at the cost of larger input image.

**Table 6:** Sensitivity (%) of three methods.

noise	0	10%	20%	30%	40%	50%	60%
Correlator–GAC coding set– 3 × 3	100	100	100	100	99.34	97.69	86.47
Correlator–GAC coding set– 9 × 9	100	100	100	100	100	99.34	95.38
Correlator–integer coding set	99.01	98.35	97.69	95.05	88.12	42.52	0
BLAST 2.9.0+	100	98.68	54.46	3.30	0	0	0

It is worth noting that the considerable outperformance of GAC method to provide ultra-high sensitive optical correlation, over the alternative approaches, is a result of its cost-function keeping a constant number of bits 1 in each code-word. Specifically, GAC cost-function is targeted to decrease overlap noises among symbols’ code-word, so it can increase signal to noise ratio at the output of a cross-correlation system. On the other hand, constant number of bits 1 in each code-word facilitates threshold value determination to locate output peaks. Specifically, the peak value is the product of query length by the number of bits 1. In this manner, when some mutations occur within the query string, we expect the peak value reduces by the ratio of "1—mutation rate". Therefore, for query length of L, mutation rate of M, and coding set with N bits 1 in each coded symbol, the peak value can be computed by Eq (7). Of course, since coded patterns of bits 1within two non-matched encoded alphabets might overlap, the cross correlation of non-matched alphabets can result in non-zero output. Hence, to address these undesirable noises at the output, Eq (8) modifies Eq (7) to include their average value as follows.

$$Peak\;value_{zero\;for\;mutation}=L\times N\times (1-M)$$ (7)

$$NormPeak\;value_{zero\;for\;mutation}=N\times (1-M)$$ (7-1)

$$Peak\;value_{real}=Peak\;value_{zero\;for\;mutation}+L\times C\times M$$ (8)

$$NormPeak\;value_{real}=N\times (1-M)+C\times M$$ (8-1)

In Eq (8), the parameter C is defined as a specific constant for each coding set. In fact, as represented in Eq 6, the mutated nucleotide increases the peak value by an amount neither equal to N nor 0, but by a fraction of N depending on signal to noise ratio of the corresponding coding set (i.e. parameter E in GAC method), which is predictable. For example, it is equal to 1.2 for the 3 × 3 coding set, while its value for the 9 × 9 coding set equals 13.5.

To clarify the proposed formula of Eq (8), which computes the meaningful peak value of the cross-correlation output, Table 7 compares peak to L values of the cross-correlation output computed by Eq (7) and Eq (7) against normalized average values and their variance obtained from all simulation studies under various mutation rates. On the other hand, we normalize Eqs (7) and (8) by L, so that Eqs (7-1) and (8-1) are obtained, whose values are mentioned as coefficient (7) and coefficient (8) within Table 7, respectively. Also, we normalize meaningful peak values, computed in each simulation scenario of the mutation rates, by dividing them with L, and also compute their average and variance values as reported in Table 7. Considering various metrics listed in Table 7 for varying mutation rates, we can conclude that Eq (8) precisely predicts normalized peak value for each mutation rate, and hence, it can be adopted for meaningful peak detection in the proposed optical system.

**Table 7:** Cross-correlation peak to L ratio for the 3 × 3 coding set.

Mutation rate	Coefficient (7)	Coefficient (8)	Coefficient (simulation)	
			Average	Variance
0	3	3	2.996928591	0.000030826
10%	2.7	2.82	2.814457584	0.000101088
20%	2.4	2.64	2.634281527	0.000133612
30%	2.1	2.46	2.451105554	0.000355068
40%	1.8	2.28	2.269366973	0.000305616
50%	1.5	2.1	2.091384926	0.00069193
60%	1.2	1.92	1.911885424	0.001049829

Summarizing above simulation studies, we can conclude that GAC coding method facilitates automatic threshold definition at the output of a cross-correlation system to exactly locate the peaks.

According to the best of our knowledge, GAC coding method is the first coding approach leading to meaningful peaks at the output of the cross-correlation system. While for those coding methods proposed in [33], and similar works [23, 24], peak values at the output pattern cannot locate the best query match, and in some cases may cause mistake in detecting peak values. For example, according to the coding method proposed in [33], with coding set (A = 65, C = 130, G = 195, and T = 255), auto-correlation of "A" leads to peak value of 4225 (= 65 × 65), while its cross-correlation with another symbol leads to larger peak. In this manner, false-positive query matching is probable, as a result of non-deterministic threshold value for peak diagnosis. For instance, for specifying threshold values, authors in [33], simulate cross-correlation for queries with various lengths, and choose minimum peak values. However, as reported in [30], this strategy causes matching error in some cases.

#### 4.4.2 Speed comparison for a small test case

The simulation study reported in [33] compares run time of integer coding method with that of BLAST assuming 303 query sequences and a reference with 100 scenes. Total run time includes the time consumed for loading 303 query sequences, encoding them, and comparing them against 100 scene images. It is worth noting that since loading and encoding reference sequence is performed once, it is not considered for comparison. It is worth noting that although we include and report the codification time of the proposed optical sequence comparison method, they can be ignored if we store coded DNA sequences.

At first, we simulate loading and encoding 303 random sequences with a random length in the range of 50 bp to 4500 bp for 10 times, and report their average run time and its variance in Table 8.

**Table 8:** Average run time for loading and encoding 303 query sequences.

	Integer coding set [33]		3 × 3 coding set (GAC)		9 × 9 coding set (GAC)	
	Loading time	Codification time	Loading time	Codification time	Loading time	Codification time
Average (Sec)	0.213694977	4.29756985	0.210760335	5.04183531	0.228442311	13.0823206
Variance	0.000133958	0.12966862	0.000265731	0.06291734	0.000164504	0.35283883

Afterwards, run time of the proposed optical setup is computed as well. Going through all comparison scenarios, we compare each scene, chosen among 303 query scenes, and against reference scenes, we perform 306936 comparisons, which equals to the total number of pairwise comparisons among 784 scenes (303 + 481). It is worth noting that the authors in [33] compare each query scene against 100 scenes as the reference scene, while we consider the whole genome. Runtime of all those comparisons depends on the display technology of the optical setup. Specifically, for an optical system utilizing Digital Micro-mirror Devices (DMD), display switching rate approximately equals to 20 kHz (and the optical processing takes 15.3468 Sec, while with holographic disc with switching rate of 2.44 MHz [34], optical processing takes 0.1258 Sec.

Assuming that the aforementioned optical correlation setup can be fed by either of GAC or integer [33] coding method, Table 9 compares whole run time of the optical DNA comparison methods as the sum of coding and loading run time (listed in Table 9) and 0.1258 Sec, as the optical processing time. This table also compares the optical approach with BLAST, as a well-known electrical method, in term of processing speed.

**Table 9:** Run time taken to process 303 query SEQUENCES IN 100 reference scenes.

	Optical Comparison			Electrical Comparison
Run time (Sec)	Cross-correlation (integer coding set) [33]	Cross-correlation (GAC-3 × 3 coding set)	Cross-correlation (GAC-9 × 9 coding set)	BLAST 2.9.0+
Loading	0.2137	0.2108	0.2284	-
Encoding	4.2976	5.0418	13.082	0
Comparison	0.1258	0.1258	0.1258	29.0529
Total	4.6371	5.3784	13.4366	29.0529

According to above discussion, codification time and time required for loading data are performance bottlenecks of our method, nonetheless, all three optical processing approaches, mentioned in Table 9, are faster than BLAST. It is worth noting that although various display devices like SLM, DMD, and holographic disc have more coding capacity compared to SLM used in [33], for a fair comparison, we assume the same coding capacity and display space as assumed in [33]. Of course, adopting faster technologies, as well as full utilization of display space, we can reduce runtime considerably. Summarizing runtime comparison, reported in Table 9, we can conclude that adopting 3 × 3 coding set for optical DNA coding in the proposed optical setup, runtime increases by about 16% compared to [33] and reduces by more than 81% compared to BLAST. It is worth noting that according to Table 6, the proposed coding approach increases sensitivity from 0 to more than 86% (in the case of worst simulation scenario with mutation rate of 60%). In case of adopting 9 × 9 coding set, sensitivity increment is more than 95% at the cost of increased runtime.

#### 4.4.3 Speed comparison for a large test case

Notwithstanding above description of speed improvement with the proposed optical setup, as follows, we compare it with two high-speed versions of BLAST methods; i.e. HS-BLASTN and MegaBLAST. For this purpose, we consider a realistic assessment with big data used in [35] whose assumptions and methods' conditions are summarized in Table 10. To assure the generality of this assessment, alongside a reference sequence, two query sets are considered; one with short length queries and another one with long length queries. In this manner, HS-BLASTN, MegaBLAST, and the proposed method search for the query sequences within the reference sequence.

**Table 10:** Assumptions of speed comparison assessment.

Parameters	Value
Query set 1 (short queries)	Homo Sapience
	#queries = 2000000
	100 ≤ length ≤ 500 bps
Query set 2 (long queries)	Homo Sapience
	#queries = 870000
	800 ≤ length ≤ 4000 bps
Reference sequence	Genome Reference Consortium Human Build 38
	Length = 3099734149 bps
Optical setup	Scene size = 4K (4096 × 2160 pixel)
	Switching speed = 2.44 MHz
	Coding set: d = 3, N = 3, E = 0, score = 0

HS-BLAST and MegaBLAST which are multi-thread based methods ran on a Linux server with two six-core Intel Xeon E5-2620 CPUs with more than 16 GB of RAM. Their runtime for these assessments, considering various number of threads, are reported in [35] which is also shown in Table 11.

**Table 11:** Runtime (second) taken to search long and short query sequence in human genome.

Program	HS-BLASTN				MegaBLAST				Optical comparison
CPU threads	1	2	3	4	1	2	3	4	-
Data set 1 (short queries)	430	138	85	68	5384	2136	1649	1495	1.31
Data set 2 (long queries)	600	185	110	85	6680	2400	1730	1537	4.58

As noted in Table 10, in this study, CAG considers 3 × 3 coding set for sequence encoding, so as the first step of runtime estimation of CAG method, we should compute the number of nucleotides filling an input image. According to the size of each input image, i.e. 4K, this value can be obtained from Eq (9) as follows.

$$\#nucleotides\;in\;each\;scene=\left\lceil \frac{scene_{width}}{coding_{width}}\times \frac{scene_{height}}{coding_{height}}\right\rceil =\left\lceil \frac{4096\times 2160}{3\times 3}\right\rceil =982800$$ (9)

In the next step, by means of Eq (10), we compute the minimum number of input images required for coding each of query and reference sequences within the optical setup, as depicted in Fig 2.

$$\begin{array}{l} \#scenes=\left\lceil \frac{length}{\#nucleotides\;in\;each\;scene}\right\rceil \\ \to \#scenes_{reference}=\left\lceil \frac{3099734149}{982800}\right\rceil =3154\\ \to \#scenes_{shortqueries}=\left\lceil \frac{2000000\times 500}{982800}\right\rceil =1017\\ \to \#scenes_{long\;queries}=\left\lceil \frac{870000\times 4000}{982800}\right\rceil =3541 \end{array}$$ (10)

And finally, according to Eq (11), we estimate the runtime of the optical setup.

$$\begin{array}{l} searching\;speed=\frac{\#scenes_{query}\times \#scenes_{reference}}{switching\;speed}\\ searching\;speed_{short}=\frac{1017\times 3154}{2440000}=1.31\;Sec\\ searching\;speed_{long}=\frac{3541\times 3154}{2440000}=4.58\;Sec \end{array}$$ (11)

It should be noted that for an accurate runtime comparison, worst case estimation is considered for the optical processing method, considering the longest query sequences. Nonetheless, according to Table 11, the optical method, taking advantages of inherent capability of light for parallel processing leads to the best runtime compared to the fastest current methods.

### 4.5 Assessment by real applications

To emphasize the importance of optimized coding through optical processing of bio data, we address k-mers counting (i.e. sequences with length of k bps), as the main step of applications like motif finding, and setup a simulation study in two parts; a) nucleotides are coded with a random 2D pattern, where, d = 3, N = 2, E = 0, and score = 1280, and b) nucleotides are coded with 2D pattern generated with the proposed code generator (GAC), where, d = 3, N = 2, E = 0 and score = 0.

Considering two aforementioned coding approaches, we encode Homo sapiens GRCh38.p12 [36], as a reference whole genome of a humane, and feed it to the proposed optical cross-correlator to find the motifs. Addressing motif finding problem within the DNA sequence, we search for all possible motifs with the length between 1 to 4 nucleotides among the first 1260 bp of chromosomes 1 to 12. In this manner, 340 possible subsequences are searched within 12 sequences considering both coding approaches; random and optimized coding. These assumptions are also shown in Table 12. To evaluate the coding performance, Eq (12), as follows, computes average motif finding errors in both cases.

$$Relative\;error=\frac{\left|N{}_{real}-N_{find}\right|}{N_{real}}$$ (12)

where, *N*_*real*_ is the actual number of motifs within a sequence and *N*_*find*_ is the number of motifs found by the cross-correlation based motif finding approach considering either random or optimized input coding. In the following sections, we address both behavioral and optical simulations in MATLAB and ZEMAX [37] simulation environments, respectively.

**Table 12:** Assumptions of k-mer counting assessment.

Parameters	Values
Coding sets‘ features	d = 3, N = 2, E = 0, score = 1280
	d = 3, N = 2, E = 0, score = 0
Sequence access ID	Homo sapiens GRCh38.p12 the first 1260 bp of Chr 1 to 12
K-mer size	K∈N,1≤k≤4

#### 4.5.1 Behavioral simulation

**Fig 11:**
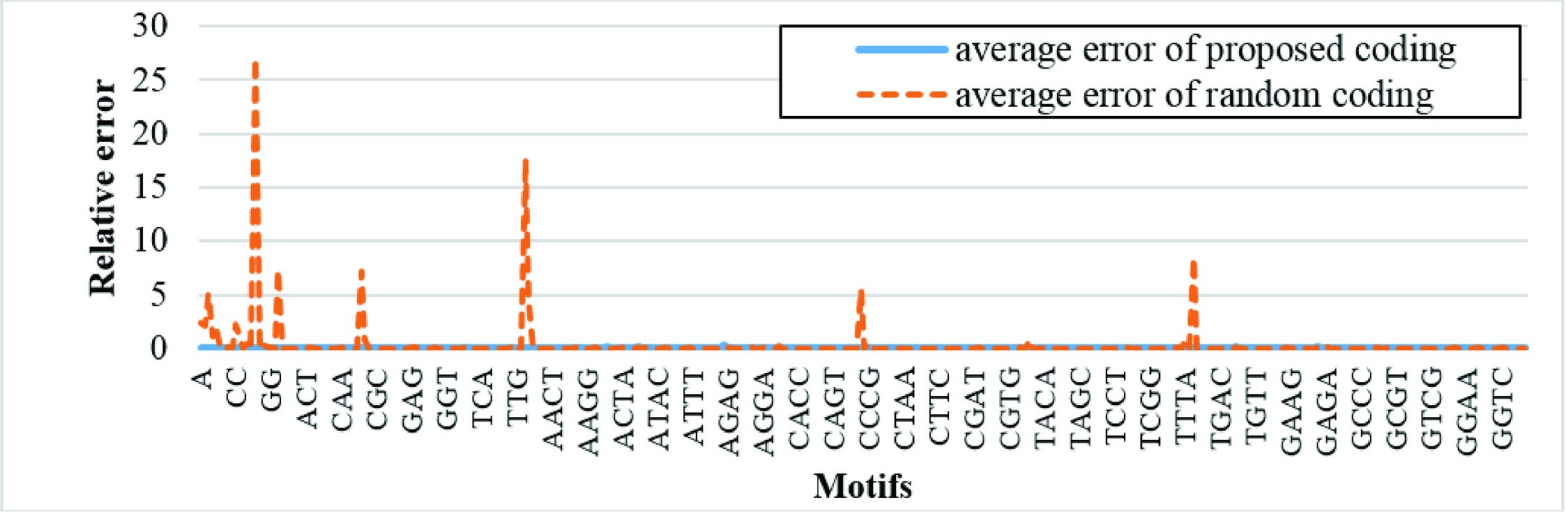
Average relative errors of cross-correlating all encoded sequences with length 1 to 4 with first 1260 bp of first 12 chromosomes of Homo sapiens GRCh38.p12 data.

In this section, the coherence theory of optic is not considered and the ideal FFT calculation is adopted. Specifically, as the simulation results illustrate, we just utilize fft2 function of MATLAB and, while not considering realistic optical parameters of the system. Fig 11 depict average motif finding errors adopting both random and optimized coding approaches.

As shown in this figure, motifs detection error is considerably reduced while adopting GAC approach, compared to the case that input data is randomly coded. Specifically, in average, motif finding relative error in the latter case (with average error equal to 34%) is about 28% more than the former one (with average error equal to 5.8%).

**Fig 12:**
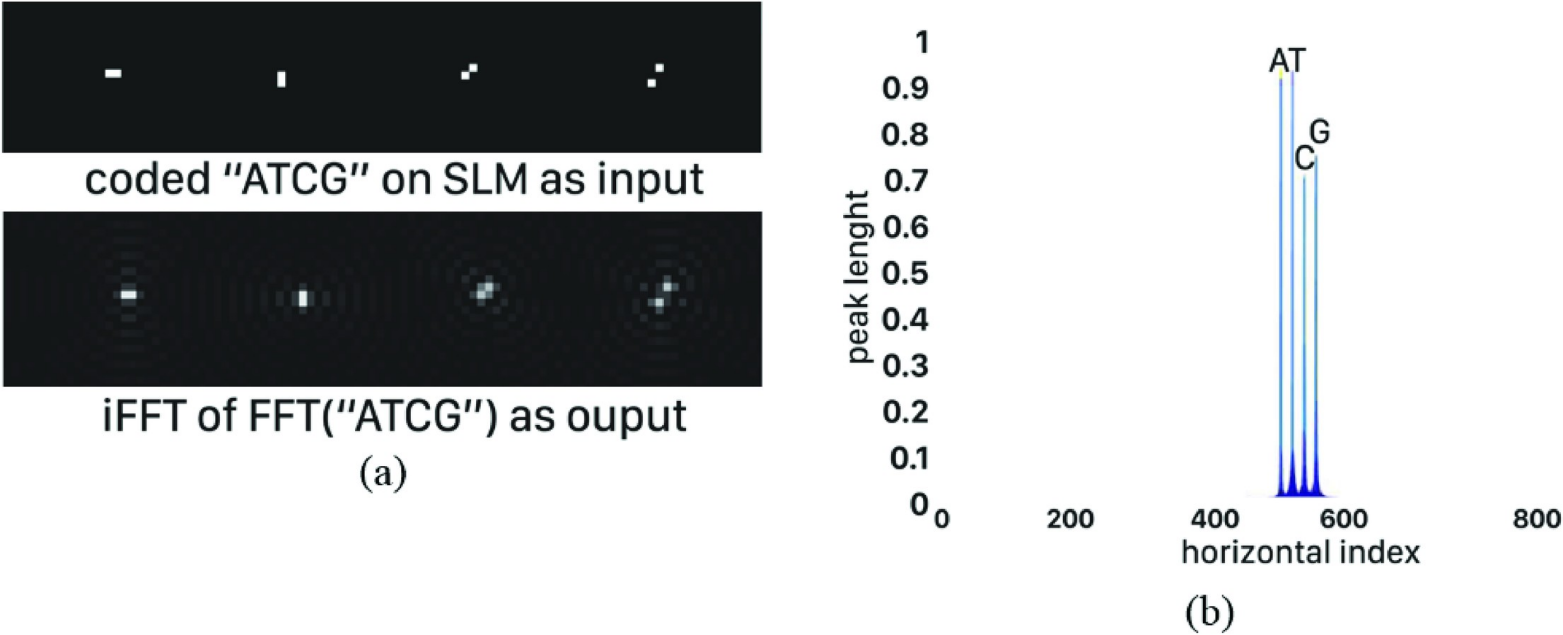
Effect of lens choice on FFT noise of sequence "ATCG" coded with coding set with d = 3, N = 2, E = 0 and score equal to 0. a) Input and output patterns, note halo created around each code at the output pattern, b) Different peak values for various coding.

It should be noted that through the simulation steps, due to the different positions of a sequence at several rows of the input image, the patterns at the end of each line may not be counted in the current method. So, a relative error, called as cutoff error, occurs. Cutoff error mostly arises when cross correlating similar patterns leads to missing string match. As an example shown in Fig 12, placement of “AA” part of the pattern “AATC” at the end of first line of the coded reference sequence and “TC” part at the beginning of the second line misses the full peak. While locating “AATC” at the third line leads to a full peak at the output of cross-correlator. In this manner, 50% relative error is reported. To address the issue, as a future work, we will propose a novel algorithm for data coding to avoid string breaks. For now, for a fair evaluation, we assume fix number of symbols columns (equal to 42 columns) throughout the simulation.

#### 4.5.2 Optical simulation

For simulating realistic condition of the optical system, we define optical parameters within the ZEMAX simulation environment. ZEMAX use pupils for characterizing imaging system. Pupils are virtual apertures that are divided into two parts; the entrance pupils collecting light from the object, and exit pupils from which the collected light exits on its way to form an image. The pupils are images of the physical elements within the optical system, known as the aperture stops, which limit the collection of light. For example, in our optical system, the lens utilized for computing inverse Fourier transform is considered as its aperture stop.

Simulating our optical system necessitates definition of three parameters; laser wavelength (λ), the distance from exit pupil (z_xp_), and diameter of exit pupil (D_xp_). These parameters are used for computing coherent cutoff frequency (f_0_), as shown in Eq (13), where, f_0_ is the frequency limitation beyond which the transfer function of the system is zero. In fact, f_0_ corresponds diffraction limit to resolution [37].

$$f_{0}=\frac{D_{xp}}{2\lambda z_{xp}}$$ (13)

In our system, z_xp_ is the focal length of lens and D_xp_ equals their diameter. We assume wavelength of HeNe laser to be 632.8 nm, z_xp_ = 4 mm cm, and D_xp_ = 80 mm for our simulation study.

Moreover, there exist other sampling parameters to be defined; physical sample interval (Δu), number of samples (M), and side length (L), whose actual values are usually chosen lower than their theoretical upper-bounds, defined as follows. Eq (14) defines the upper bound of Δu depending on the pupil parameters and laser wavelength. As shown in this equation, maximum value of Δu is obtained assuming ideal system condition. Using this equation and the relation L = MΔu, we can calculate the image dimensions to be used in the optical system by Eq (15). For simulating the realistic condition, Δu and L value are considered 5% less than their upper-bound to consider the system error.

$$\Delta u\leq \frac{\lambda z_{xp}}{2D_{xp}}$$ (14)

$$L\leq M\frac{\lambda z_{xp}}{2D_{xp}}$$ (15)

**Fig 13:**
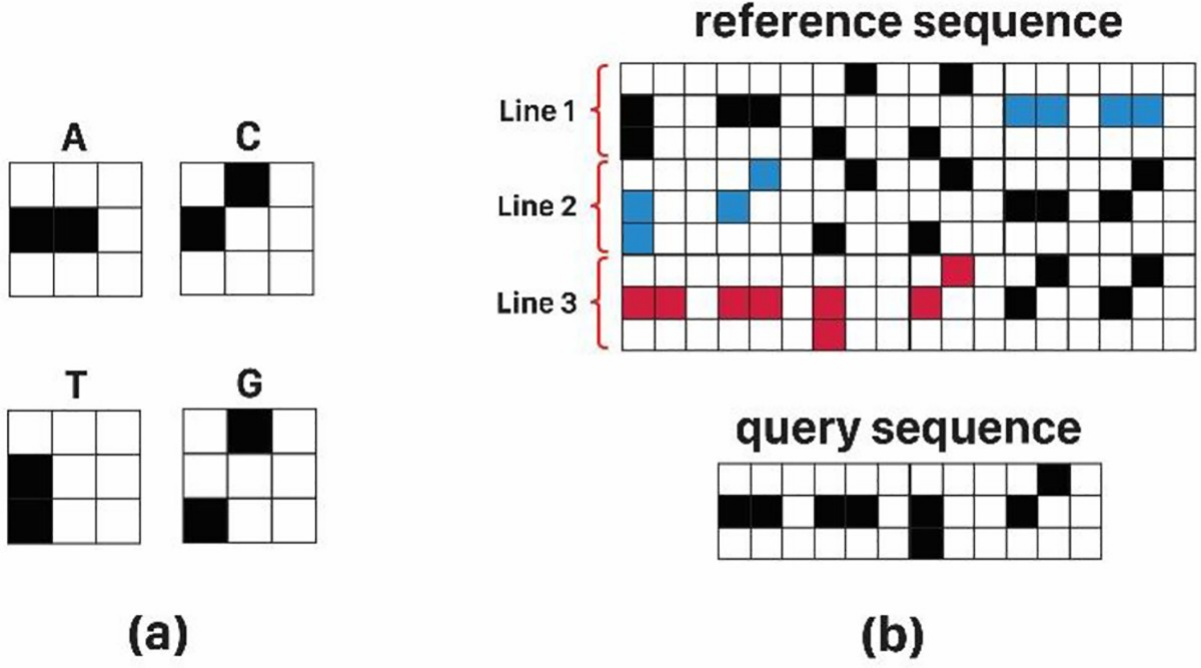
Example of cutoff error; a) coding set, b) "TAGGAATCGGACAATCCC" as the reference sequence is splitted into 3 lines with 6 codes, while "AATC" is th equery sequence. End of line 1 and begining of line 2 contain query sequence which is breaked from middle and it cannot be detected by the cross-correlation process.

Values of aforementioned parameters considerably impact system resolution and output noise of the cross-correlator. This effect is well observable in Fig 13A; this image is the result of the inverse Fourier of Fourier transform of sequence "ATCG", coded by coding set generated by GAC, with d = 3, N = 2, E = 0, and score = 0. As can be seen, a halo is created around each of the nucleotide code depending on their shape, which indeed generates undesirable noise at the output. According to Eqs (13) to (15), the greater ratio of the lens diameter to its focal length, which represents the lens's ability to absorb light, reduces halo in output. However, this feature is limited by manufacturing technology of the lens. On the other hand, according to Fig 13B, in addition to the produced noise around each optical code, the height of their peaks can also vary from the expected value depending on the shape. Specifically, in Fig 13B, the peak values of cross-correlating various nucleotides were expected to be equal but some diagonal structures (occurs for C and G nucleotide codings) within the coded pattern have reduced their peaks values.

**Fig 14:**
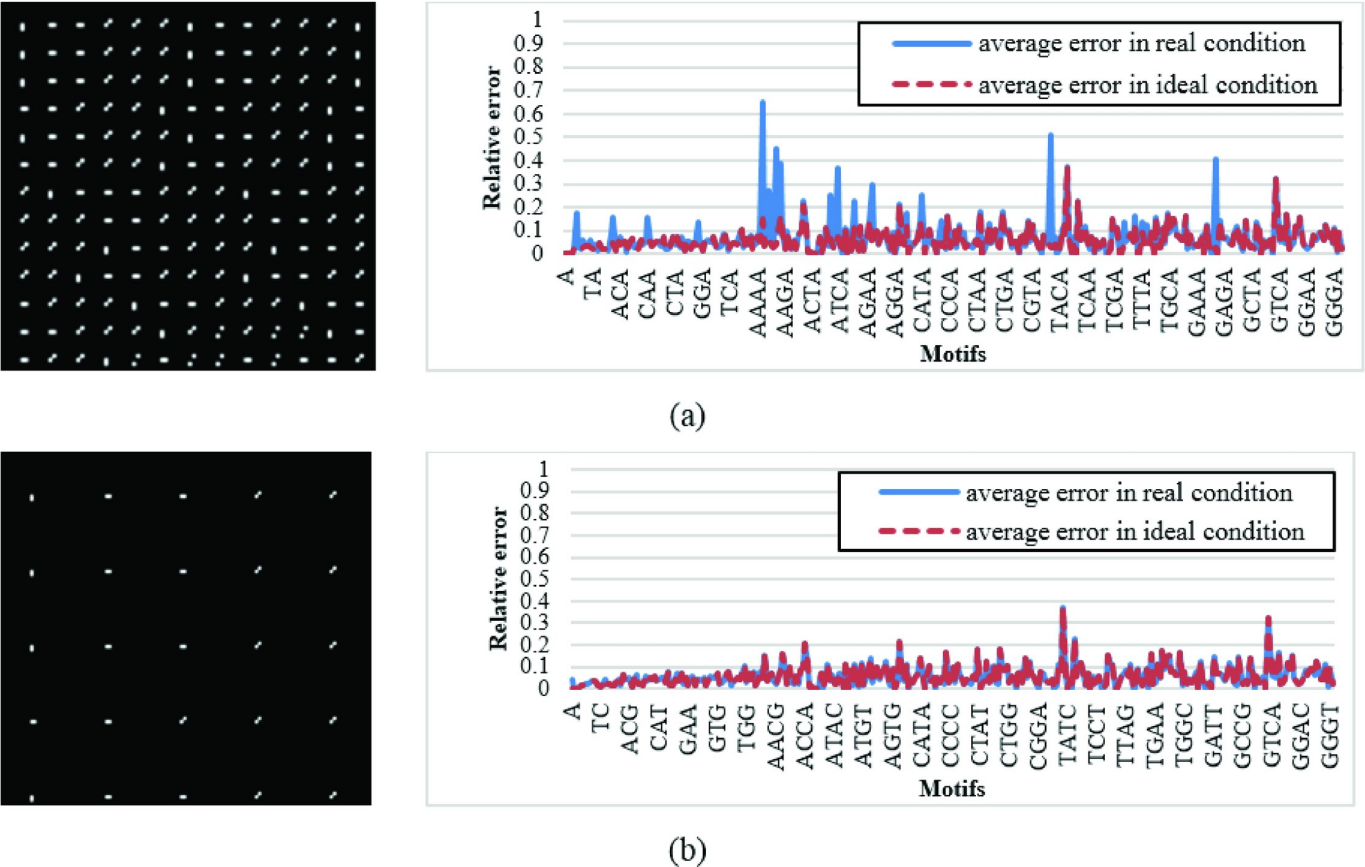
Average relative errors of cross-correlating all motifs with length 1 to 4 with first 1260 bp of first 12 chromosomes of Homo sapiens GRCh38.p12 data (sequences are encoded using coding set with d = 3, N = 2, E = 0 and score equal to 0, while each line of SLM cosist of 42 columns). On the left, a part of the coded reference sequence is shown, while on the right, average relative errors resulted from optical simulation is compared with that of bahaviolral simulationconsidering free boundary of width a) 2 pixels and b) 10 pixels around each nocletide code.

Unfortunately, the difference between peak values of cross-correlating various nucleotides, as well as, their surrounding haloes arise serious problems for recognition of full peaks. Specifically, for the purpose of pattern matching, we should specify a threshold value, while the output values greater than it represent full peaks. However, according to above discussion, the output noise prevent determining threshold value. As a key solution to this problem, we propose inserting free space between adjacent coding patterns of consequent nucleotides on the SLM. It should be noted that longer distance length reduces coding efficiency while improving output noise. To illustrate this trade-off, we simulate a realistic optical system assuming two coding scenario; a) each 2D optical code devotes a free boundary, with the width of two pixels, around itself, and b) each 2D optical code devotes a free boundary, with the width of 10 pixels, around itself. It should be noted that in both cases the same coding set of 3 × 3 codes is adopted, and 42 columns are considered for each symbol line in SLMs. The coding pattern and the corresponding cross-correlation results are shown in Fig 14.

As depicted in Fig 14, larger free space among consequent codes improves relative error. Specifically, average relative error in the case of two-pixel-width boundary around optical codes equals 7.2% (as shown in Fig 14A), while this value reduces to 5.8% (as shown in Fig 14B) in the case of 10-pixel-width boundary around optical codes. Moreover, larger free space around optical codes simplifies threshold value determination. As an important point in Fig 14B, both ideal and real case matches behave similarly with an average relative error equal to 5.8%. As discussed in previous Section, this amount of relative error, named as cutoff error, arises from the string breaks at the end of line. However, as depicted in Fig 14B, considering a free boundary, with the width of 10 pixels, around each nucleotide code leads to the same simulation results for realistic and ideal system simulations.

**Fig 15:**
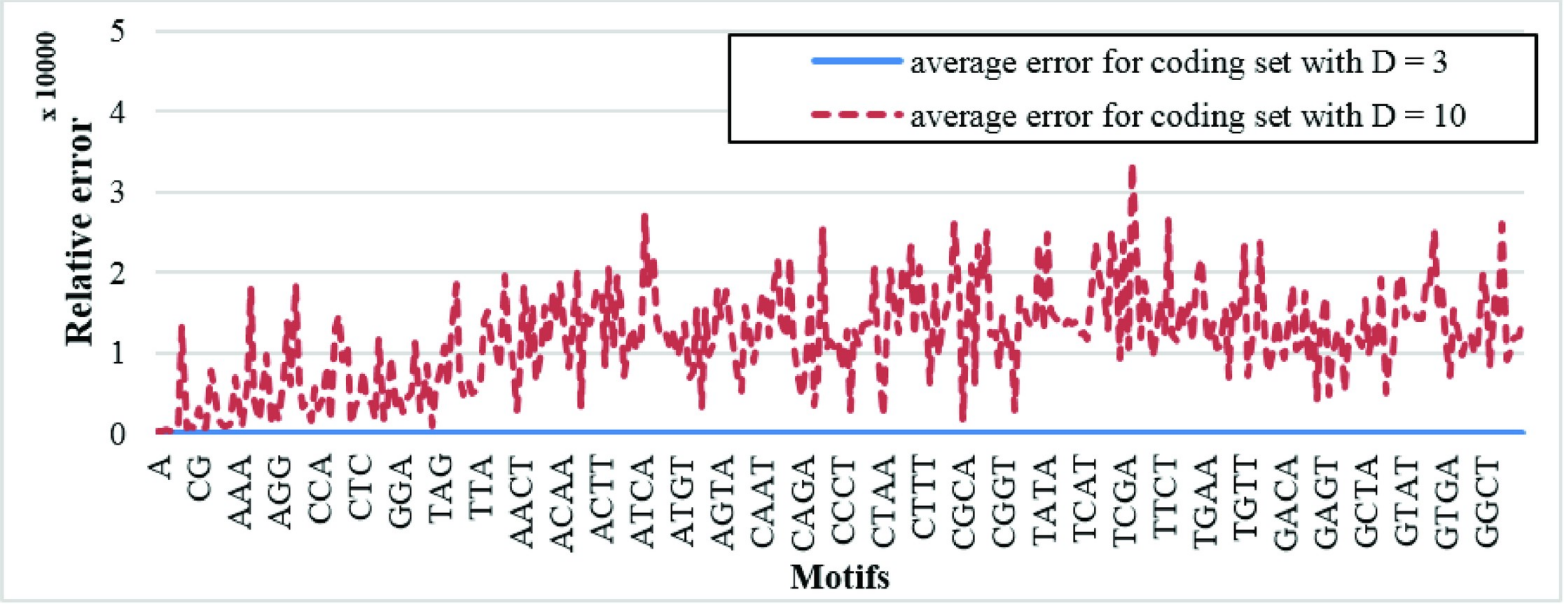
Average relative errors of cross-correlating all motifs with length 1 to 4 and first 1260 bp of first 12 chromosomes of Homo sapiens GRCh38.p12 data (sequences are encoded by coding set with d = 10, N = 30, E = 12, and zero-score, each line of SLM cosist of 42 columns and extra 2 pixels are added around each coding). Result of optical simulation is compared with ideal simulation.

Finally, to investigate the impact of code size on the output noise, we also simulated the optical system assuming 2D optical codes with attributes d = 10, N = 30, E = 12, and zero-score to As illustrated by the simulation results in Fig 15, large optical pattern, in addition to reducing coding efficiency, complicates threshold selection, and thus, increases the relative error.

## 5. Conclusion and future works

Sequence alignment is one of the important bioinformatics tools with a lots of design challenges such as speed, accuracy, and length limitation of comparable sequences. Utilizing optical technology and applying optical pattern recognition techniques, such as cross-correlation, we are able to address the aforementioned issues. For this purpose, it is necessary to design different levels of system hierarchy including input data coding, as well as, optical implantation. In this paper, we investigate the effect of optimized input coding on the output noise, and provide a solution for generating customized codes for textual data (such as DNA). For this purpose, we present a genetic algorithm-based code generator, called GAC, which is capable of generating 1D and 2D optical codes for various coding sizes under varied conditions. To evaluate the impact of these codes on the output of a pattern recognition system, we perfomed various simulations on Homo sapiens genome (GRCh38.p12) and Salmonella genome (NC_003198.1 in NCBI) to show that GAC method improves all four reported metrics (i.e. Sensitivity, Specificity, Error, and Exactitude) to improve pattern detection quality. To the extent that using GAC coding set with size 3 × 3 in an optical correlator increases sensitivity and runtime speed more than 86% and 81%, respectively, in high-mutated genome state (60% mutation) in comparison to BLAST method. Of course, this trend is preserved by increasing size and relative threshold value as the inputs of GAC method. Specifically, our simulation results confirm that optical implementation of GAC coding set with size 9 × 9 increases sensitivity and runtime speed by more than 95% and 50%, respectively, compared to BLAST method.

Finally, we would like to mention that although the proposed cross-correlation-based optical method is customized for genome comparison, without loss of generality, adopting a compatible encoding method, it can be utilized for cross-correlation based text analysis. Hence, development of the proposed optical encoding and comparison approach, as the first step of an ultra-fast sequence analysis method, is not stopped here; it will be improved and adopted to a wide range of pattern detection problems. Moreover, as the future works, we plan to improve speed of GAC method, as well as accuracy of the proposed optical cross-correlation setup, and customize it for essential applications, such as SNP discovery studies and comparison of eukaryote whole-genome sequences. And finally, more details of the optical processing setup will be released.

## Supporting information

S1 File GAC method. (DOCX)

## Abbreviations

SA: Sequence Alignment
SLM: Spatial Light Modulator
DP: Dynamic Programing
GAC: Genetic Algorithm based Code generator
CMF: Classical Matched Filter
SOM: Sum Of Multiplication
FFT: Fast Fourier Transform
