# Supplementary material for: Optical pattern generator for efficient bio-data encoding in a photonic sequence comparison architecture
Source: PLoS One. 2021 Jan 15;16(1):e0245095. doi: 10.1371/journal.pone.0245095 (PMC7810328; doi:10.1371/journal.pone.0245095)
Supplement: S1 File — (DOCX) [file pone.0245095.s001.docx]

**Article type: Full Article**

Title

Optical Pattern Generator for Efficient Bio-data Encoding in a Photonic Sequence Comparison Architecture

Saeedeh Akbari Rokn Abadi ^1^, Negin Hashemi Dijujin ^2^, Somayyeh Koohi ^3*^, Zahra Kavehvash^4^

^1, 2, 3^ Department of Computer Engineering, Sharif University of Technology, Tehran, Iran

^4^ Department of Electrical Engineering, Sharif University of Technology, Tehran, Iran

***Correspondence:**

Somayyeh Koohi, Department of Computer Engineering, Sharif University of Technology, Azadi Ave., Tehran, IRAN, P.O. Box: 11155-9517.

E-mail: koohi@sharfi.edu

# Cross-correlation mathematics

In signal processing, cross-correlation is a method for measuring the similarity among two series of functions with relative displacement. This method is capable of applying on 1D, 2D, and 3D signals for any kind of pattern recognition application. Given that in the here targeted applications the inputs are in the form of one-dimensional (1D) and two-dimensional (2D) spatial patterns, 1D and 2D cross-correlation is considered in the following.

The cross-correlation function between two 1D functions *r_1_(x)* (reference) and *q_1_(x)* (query) and between two 2D functions *r_2_(x, y)* (reference) and *q_2_(x, y)* (query), are defined as follows:

| (1-a) | $c_{1D}=r_{1}\left( x \right)*q_{1}\left( x \right)=\iint r_{1}\left( \varepsilon\right)q_{1}^{*}\left( \varepsilon-x \right)d\varepsilon$ |
| --- | --- |
| (1-b) | $c_{2D}=r_{2}\left( x, y \right)*q_{2}\left( x,y \right)=\iint r_{2}\left( \varepsilon,\delta\right)q_{2}^{*}\left( \varepsilon-x,\delta-y \right)d\varepsilon d\delta$ |

In these equations, *c_1D_* and *c_2D_* are the results of one-dimensional and two-dimensional correlations, respectively, and * represents the complex conjugated symbol. According to *Figure S1*, the correlation function, either 1D or 2D, takes its maximum value when an exact replica of the query exists in the reference sequence.


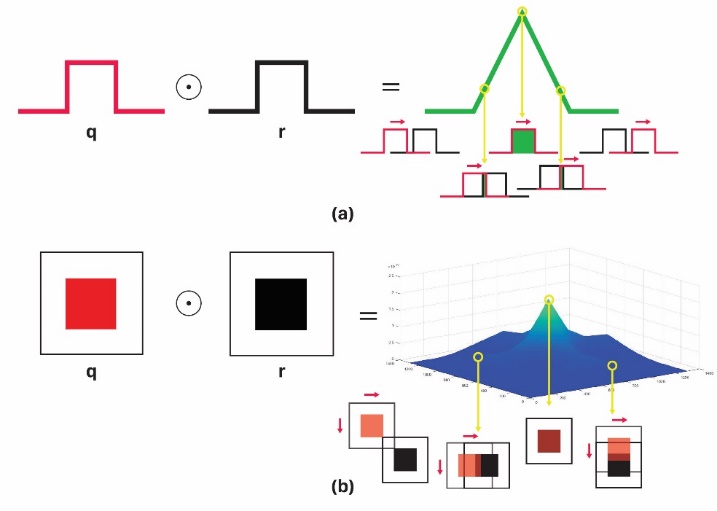


Figure S1 a) 1D cross-correlation, b) 2D cross-correlation

The cross-correlation operation could also be discussed in the frequency domain. In the frequency domain, the cross-correlation relation will be converted to a simple multiplication of input Fourier patterns:

| (2-a) | $TF^{-1}\left\{ c_{1D}(x) \right\}=R\left( u \right)Q^{*}(u)$ |
| --- | --- |
| (2-b) | $TF^{-1}\left\{ c_{2D}(x,y) \right\}=R\left( u,v \right)Q^{*}(u,v)$ |

Where *R* and *Q*, represent the Fourier transform *r* and *q*, in the frequency domain, respectively. *Q** function also shows a classical matched filter (CMF) or a mask.

# GAC algorithm parameters analysis

This section investigates how different choices of code features; i.e. d, N, and E, affect output noise of the cross-correlation.

## Case study: Coding impact on output noise reduction

### Effect of coding optimization

First of all, to illustrate how triple features, d (code size in each dimension), N (percentage of bit 1 within the code), and E, affect three types of noise (i.e. overlap noise, system noise, neighbor noise), we examined an example. In this example, a set of 2D Zero-scored codes with d = 10, E = 10, and N = 40 are compared to another set of codes with same coding patterns for C, G, and T, but a different one for A which causes Overlap noise to increase. This coding difference among two sets maximizes Overlap noise, and leaves no tolerance for neighbor noise. To clarify the coding impact, “AAAAA” and “AAAAAAAAAAACAAA” are considered as the query and reference sequences, respectively. Although this example demonstrates only two short sequences, it emphasizes the critical role of zero-scored sets and threshold values in noise tolerance. Cross-correlation result and parameters affecting its peaks are depicted in Figure S2. Meanwhile, 2D codes of the letters A and C, which is used to encode the reference and the query sequence in two experiments, are shown in the corresponding Sections in Figure S2. As discussed in Section 3.1 of paper, by cross-correlating these two sequences, seven separate peaks with maximum value should be seen, as is clearly shown in Figure S2. a. The latter key property stems from the more distinct coding, while a different coding set leads to undetectable peak values in Figure S2.b because of creating more overlap noise.


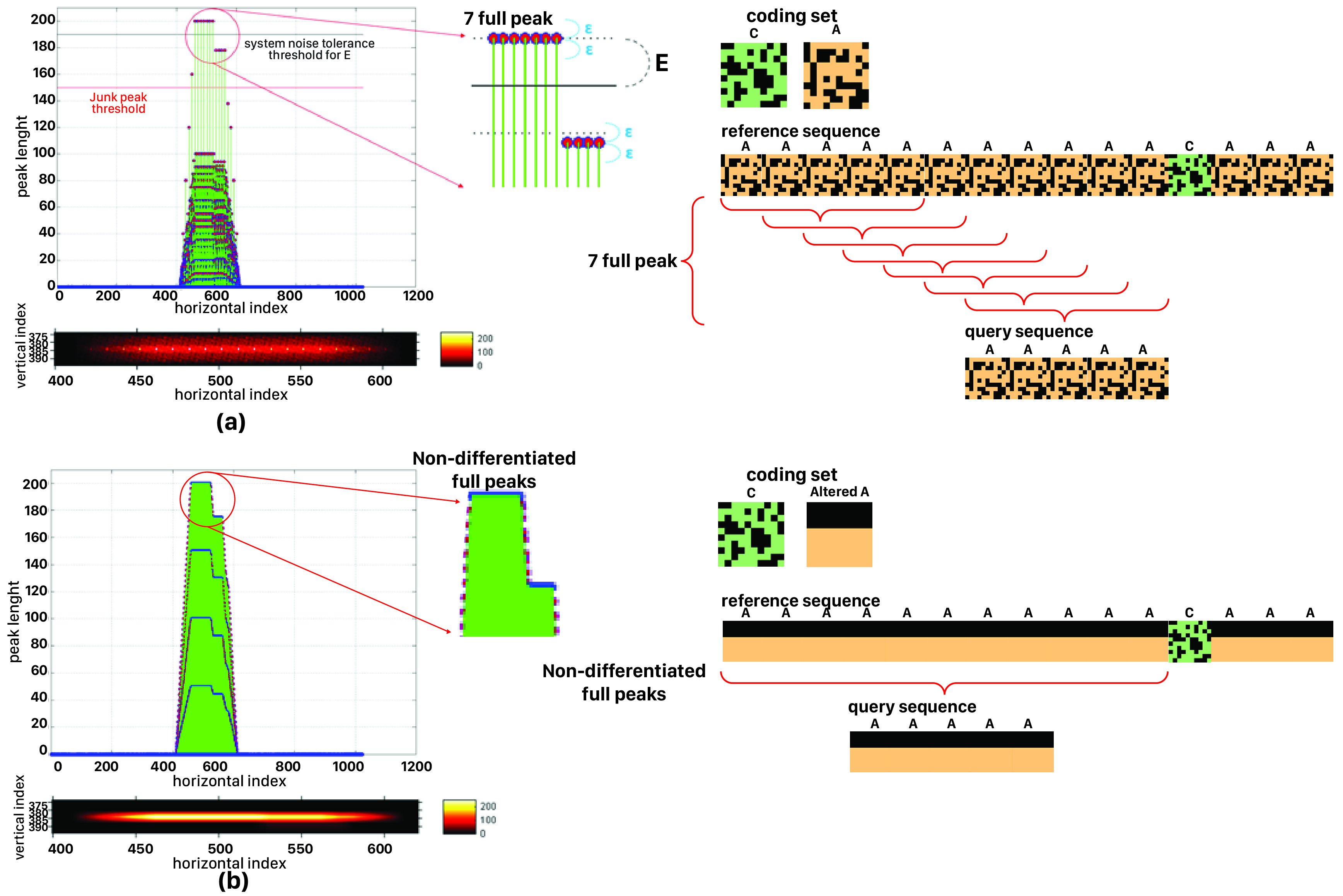


Figure S2 Cross-correlation output of AAAAA and AAAAAAAAAAACAAA, a) Distinct peaks in the output using a zero-scored set of codes with d = 10, N = 40, and E = 10; b) Unclear peaks using same coding pattern for C, G, T, but different one for A.

### Effectiveness of each coding parameters

Each of the triple parameters d, N, and E is effective on the output noise of the optical system. In the following, we plan to examine three sets of twin coding sets, each of these two coding sets are different in just one of three parameters. In all of these examples, we chose two strings "AAAAA" and "AAAAAAAAAAACAAA" for coding and compare their cross-correlation outputs.

In the first example, one of the coding sets has d = 10, N = 50, and E = 12 and another set has d = 13, N = 50, and E = 12. Figure S*3*.a refers to the coding set with d = 10 and its usage and Figure S*3*.b is related to coding set with d = 13 and its usage. As expected, the magnitude of d makes the full peak more distinct due to the increased distance between them. Also, since N is the same in both sets of codings, the magnitude of the full peak is the same, but because of the space for generating more varied patterns, one mutation can create a higher peak difference.

| 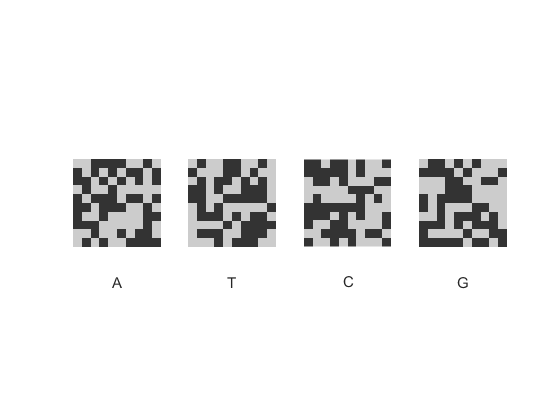  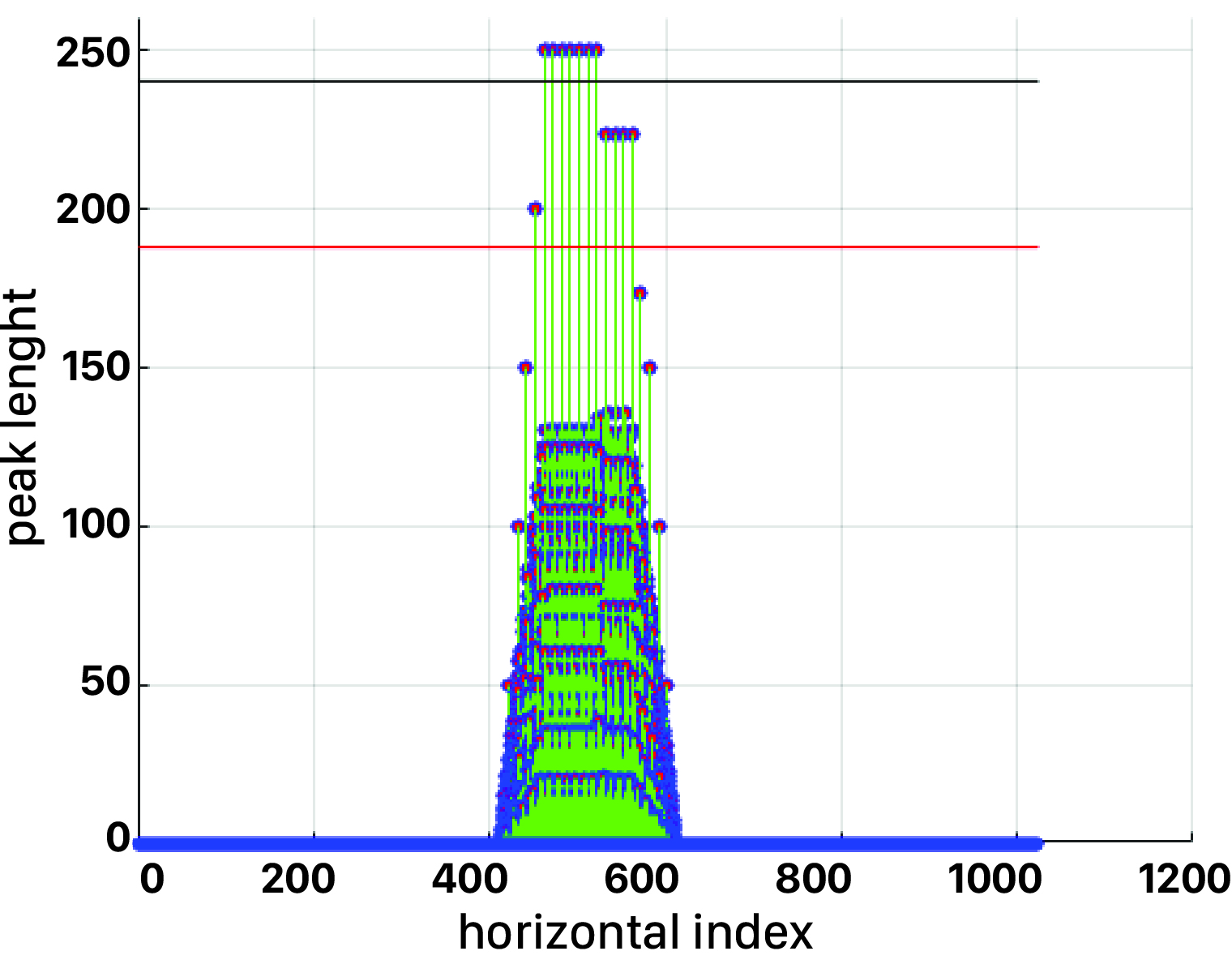  (a) | 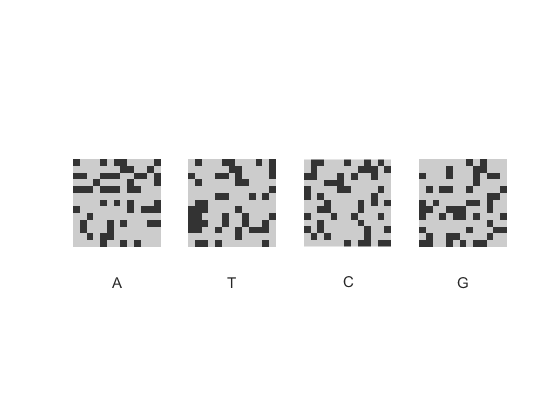  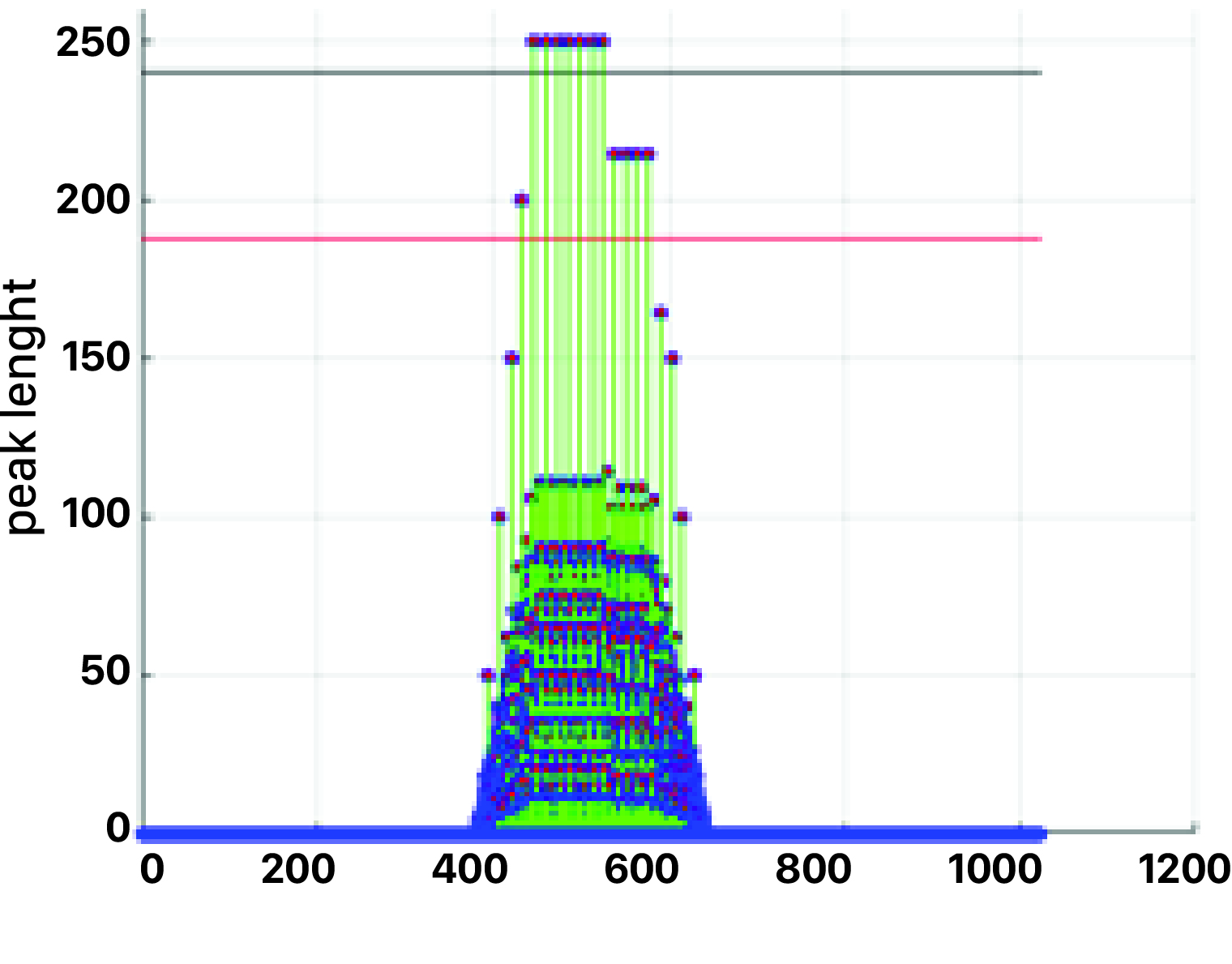  (b) |
| --- | --- |

Figure S3 Cross-correlation output of "AAAAA" and "AAAAAAAAAAACAAA" for two values of d parameter a) coding set with d = 10, N = 50, and E = 12 and cross-correlation output; b) coding set with d = 13, N = 50, and E = 12 and cross-correlation output

In the second example that N is varied, one of the coding sets has d = 13, N = 84, and E = 25 (Figure S4) and another set has d = 13, N = 101, and E = 25 (Figure S4.b). According to Figure S4.a and Figure S4.b, the magnitude of N has a direct effect on full peak height. But on the other hand, it also has an effect on increasing the output noise (such as overlap noise); so larger E must be considered for controlling this noise.

| 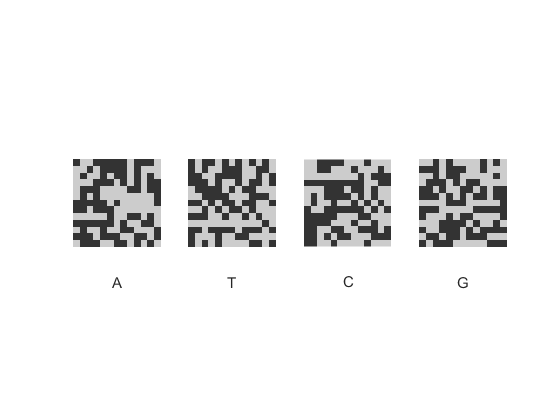  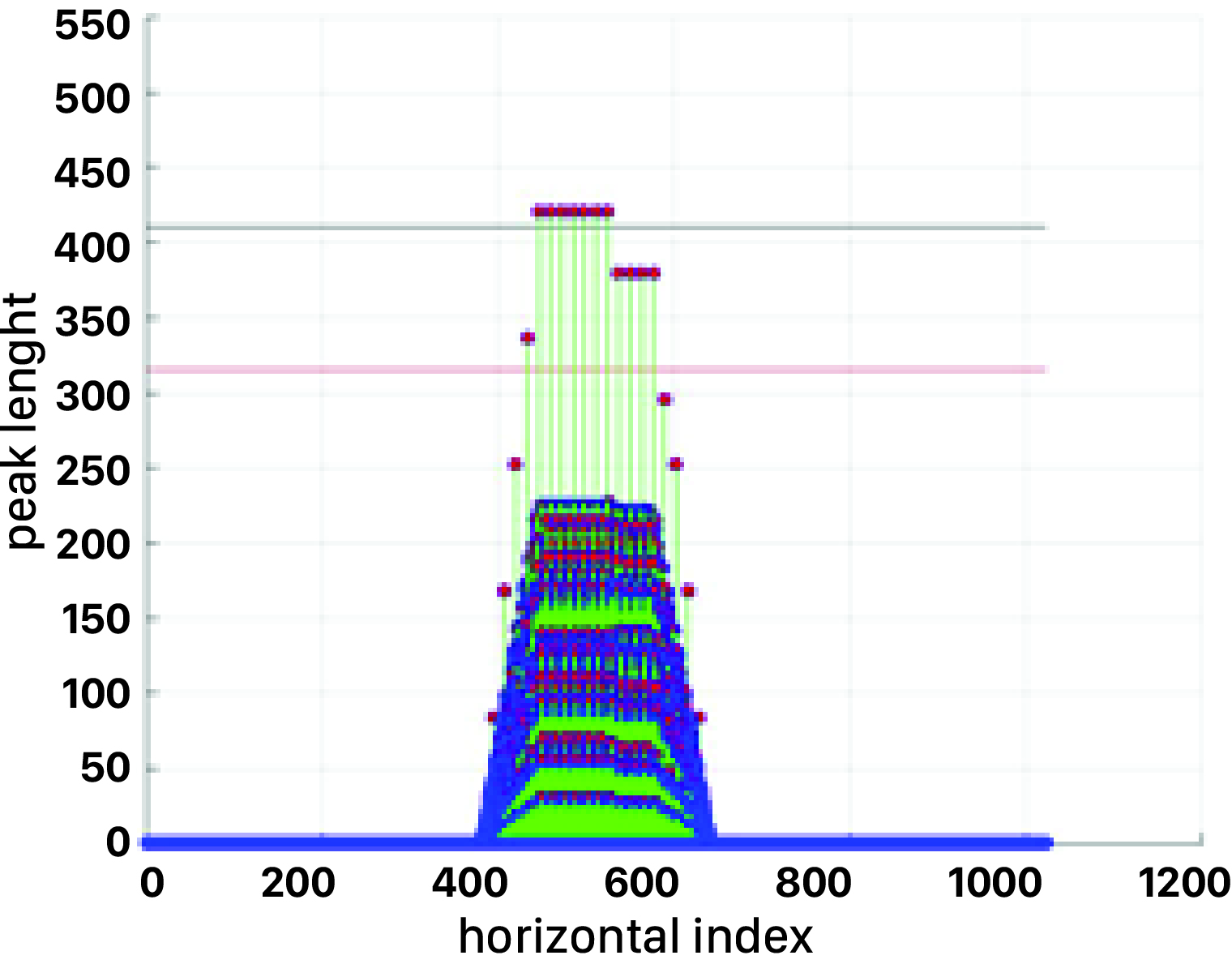  (a) | 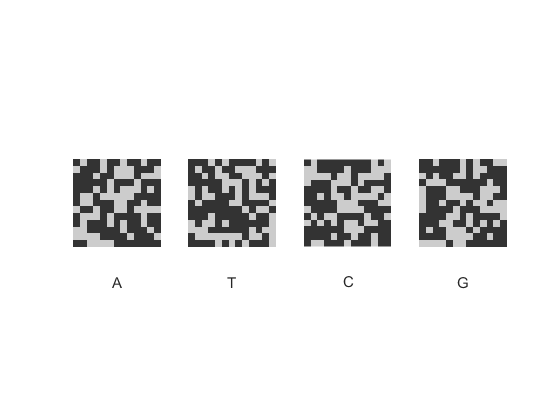  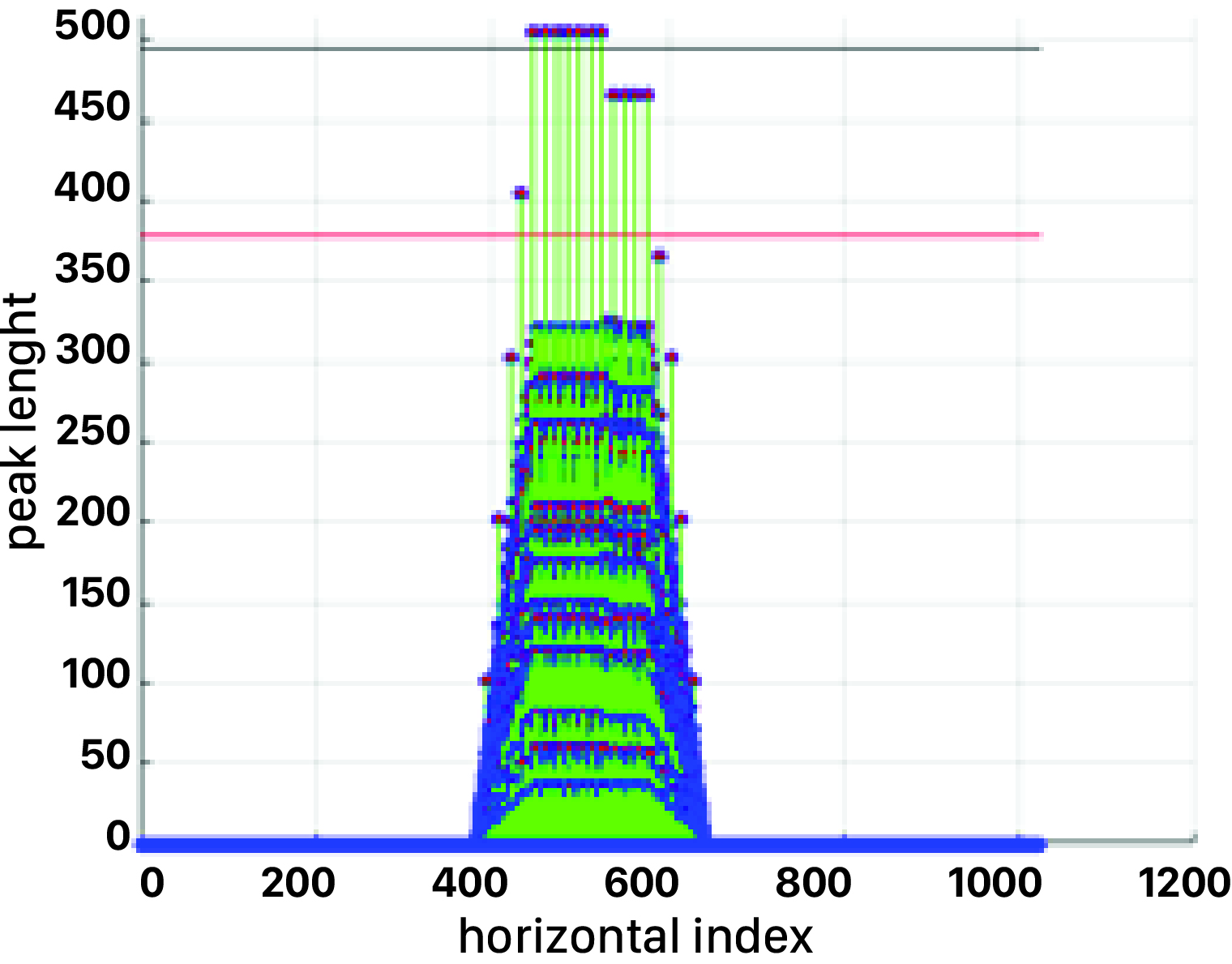  (b) |
| --- | --- |

Figure S4 Cross-correlation output of "AAAAA" and "AAAAAAAAAAACAAA" for two values of N parameter; a) coding set with d = 13, N = 84, and E = 25 and cross-correlation output; b) coding set with d = 13, N = 101, and E = 25 and cross-correlation output‏‏

Finally, the last example contains two sets with different E where one of them has d = 12 N = 57, and E = 14 (Figure S*5*.a) and another one has d = 12, N = 57, and E = 22 (Figure S*5*.b). According to Figure S*5*.a and Figure S*5*.b, the height of peaks and space between them are the same in two examples, but there are some differences in noise accumulation in their output. As described in the definition of E, more value of E increases the distance between noise and peaks, and this event is visible for the biggest E in Figure S*5*.b.

| 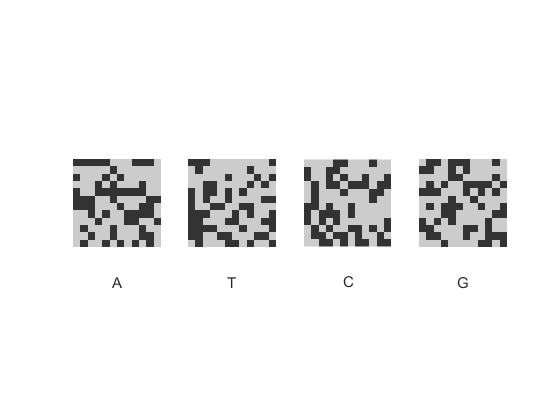  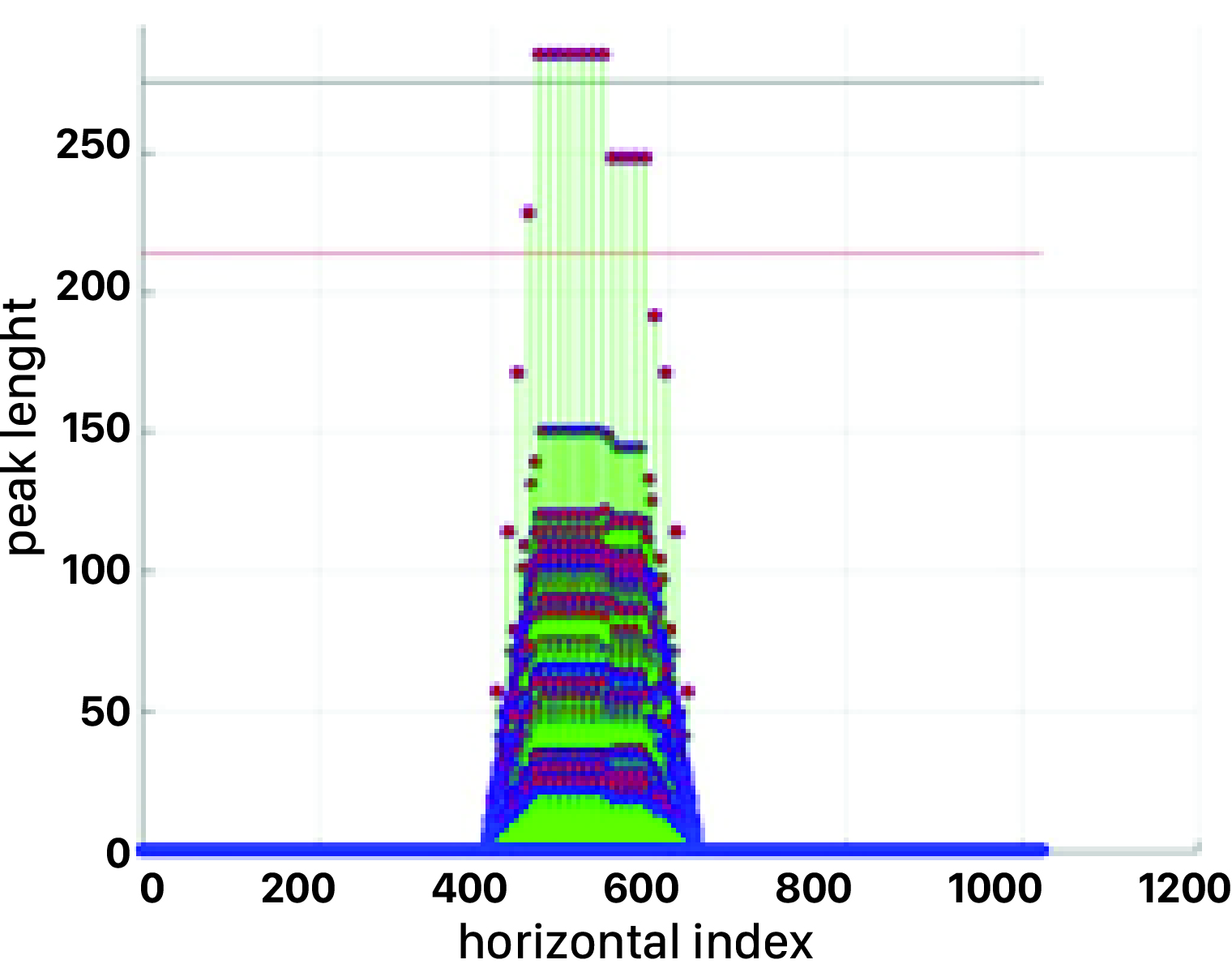  (a) | 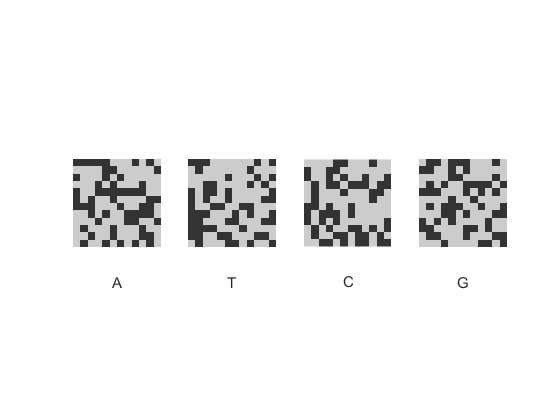  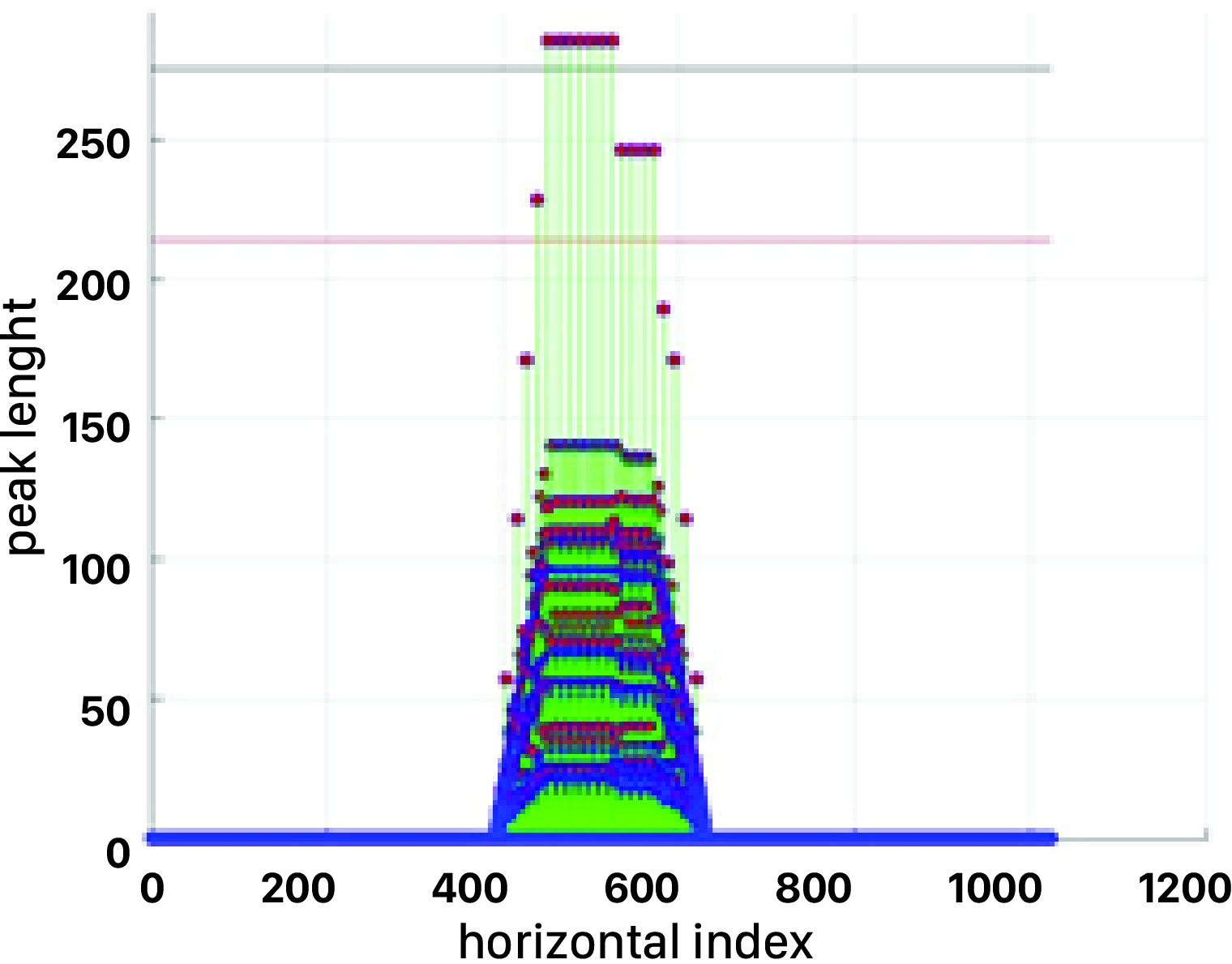  (b) |
| --- | --- |

Figure S5 Cross-correlation output of "AAAAA" and "AAAAAAAAAAACAAA" for two values of E parameter; a) coding set with d = 12, N = 57, and E = 14 and cross-correlation output; b) coding set with d = 12, , N = 57, and E = 22 and cross-correlation output

## Zero-score coding sets distribution

To investigate the effectiveness of optimizing d, N, and E on the coding score, we run GAC method for both coding modes; 1D and 2D. Running conditions congruent with 2D codes are listed as:

$d\in\{3, 4, . . . , 15\}$,

$N\in\{\left\lfloor i \times d\times d \right\rfloor|\forall d, i = 5\%, 10\%, 15\%, 20\%, 25\%, 30\%, 40\%, 50\%, 60\%, 70\%\}$,

and $E\in\{\left\lfloor5\%\times i\times N \right\rfloor|\forall d, N, i = i in Z, 0 < i < 15\}$.

In the case of 1D codes, similar parameters are assumed except that $d\in\{3, 4, . . . , 50\}$, given runtime of 1D code generator is lesser than that of 2D one. Moreover, it should be noted that total numbers of produced codes are 4191628 and 753104 in the cases of 1D and 2D code generation, while 158 and 275 codes have zero score for each mode, respectively. Considering all generated codes, best ratio of E to N equals 0.41667 with d = 50 and N = 12 in the case of 1D codes, and 0.517241 with d=14, N=29, and E=15 in the case of 2D codes.

To quantify the impact of different code features on the accuracy of cross-correlation output, we define new metrics, as follows, whose values, for 1D and 2D coding modes, are calculated in Figure S6, Figure S7, Table S*I*, and Table SII.

1. Relative threshold

Definition 2 - Relative threshold: Since the absolute value of peak acceptance threshold (E) depends on the size and number of 1 bits of each coding set, the relative threshold is defined as the ratio of E to N to eliminate this dependency.

By defining relative threshold, all codings with any values of triple d, N, and E become comparable and we can depict generated zero-score codings according to three dependent variables d, N and relative threshold in Figure S6 (in the case of 1D coding) and Figure S7 (in the case of 2D coding). These figures illustrate the distribution of optimal codings according to their properties. As follows, we explore coding choices discussing Figure S6, Figure S7, Table S*I*, and Table SII.

Larger value of relative threshold is the more stringent conditions for generating the coding sets with lesser noise, while tolerating more neighbor noise. The later property stems from the fact that large E/N values limit the noise level to the maximum ratio of (N-E)/N with respect to the full peak, regardless of the input patterns. In this manner, we extract the maximum amount of relative threshold achieved for each size of codes in Table S*I*, and Table SII. According to these tables, the larger code size facilitates generation of distinct coding sets with higher relative threshold.

1. Valuable N

Definition 3 - Valuable N: It is defined as the value of N (as the percentage of bit 1 in a coding set) which generates the largest number of zero-score codings.

According to Figure S6.b and Figure S7.b, the highest number of zero-score codings is created by average values of N equal to 36.9% and 29%, in the case of 1D and 2D coding modes, respectively. For more clarity, second rows of Table S*I*, and Table SII, respectively, represent Valuable N for each size d. As shown in these tables, either a small number (N = 5%) or a large number of 1 bits (N = 70%) reduces the production probability of distinct patterns.

1. Number of zero-score per d

As shown in Figure S6.a and Figure S7.a, and highlighted in Figure S6.c and Figure S7.c, zero-score coding sets are mostly produced for the large sizes of coding sets. Specifically, the latter figures depict distribution of generated zero-score codings for various sizes of coding sets in the case of 1D and 2D coding. According to the later property of large coding sets, as we discussed in Section 4.1 of paper, increasing the number of probable coding sets by choosing larger values of d, we can reduce convergence time for generating optimum coding set at the cost of increasing population generation time. In all, we can conclude that there exists a trade-off between d, evolution time and population generation time, while the final coding choice depends on the system limitations.

Table SI Statistics of 1D zero-scored coding sets

| size | 10 | 15 | 20 | 25 | 30 | 35 | 40 | 45 | 50 |
| --- | --- | --- | --- | --- | --- | --- | --- | --- | --- |
| Max relative threshold | 0 | 0.167 | 0.222 | 0.25 | 0.300 | 0.333 | 0.333 | 0.364 | 0.417 |
| Valuable N | 35% | 45% | 55% | 35% | 35% | 35% | 25% | 25% | 25% |
| Number of zero-score | 2 | 3 | 4 | 4 | 6 | 7 | 9 | 11 | 13 |
| Total produced encodings | 443847 | 91783 | 68770 | 71094 | 51396 | 42307 | 33882 | 29380 | 26049 |

Table SII Statistics of 2D zero-scored coding sets

| size | 3 | 4 | 5 | 6 | 7 | 8 | 9 | 10 | 11 | 12 | 13 | 14 | 15 |
| --- | --- | --- | --- | --- | --- | --- | --- | --- | --- | --- | --- | --- | --- |
| Max relative threshold | 0 | 0.167 | 0.200 | 0.286 | 0.333 | 0.368 | 0.417 | 0.500 | 0.500 | 0.418 | 0.485 | 0.517 | 0.500 |
| Valuable N | 30%,  40% | 40% | 20%, 40% | 20% | 20%, 30% | 30% | 15% | 10% | 15% | 15% | 20% | 15% | 10% |
| Number of zero-score | 2 | 3 | 6 | 8 | 12 | 17 | 21 | 34 | 29 | 33 | 34 | 37 | 39 |
| Total produced encodings | 397420 | 144545 | 73683 | 55155 | 29849 | 16991 | 10487 | 13212 | 6704 | 4380 | 388 | 190 | 100 |


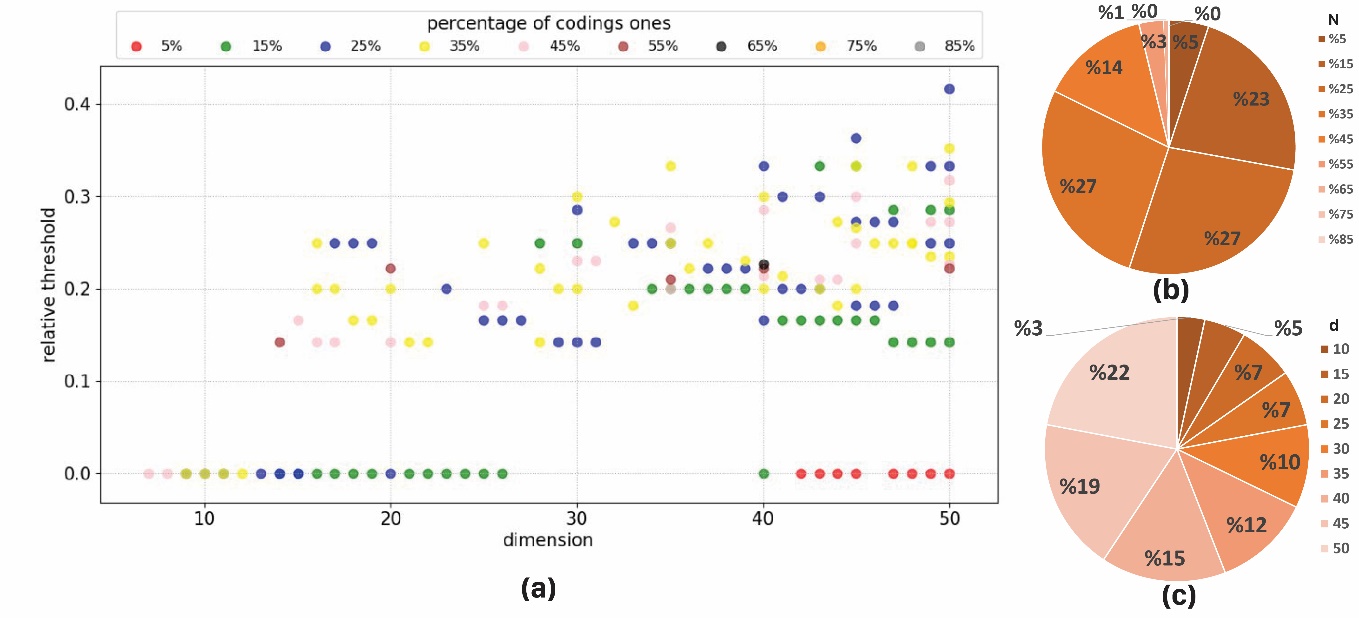


Figure S6 a) Generated zero-score 1D codings for various values of triple d, N and relative threshold, b) zero-score 1D codings distribution according to N, c) zero-score 1D codings distribution according to d


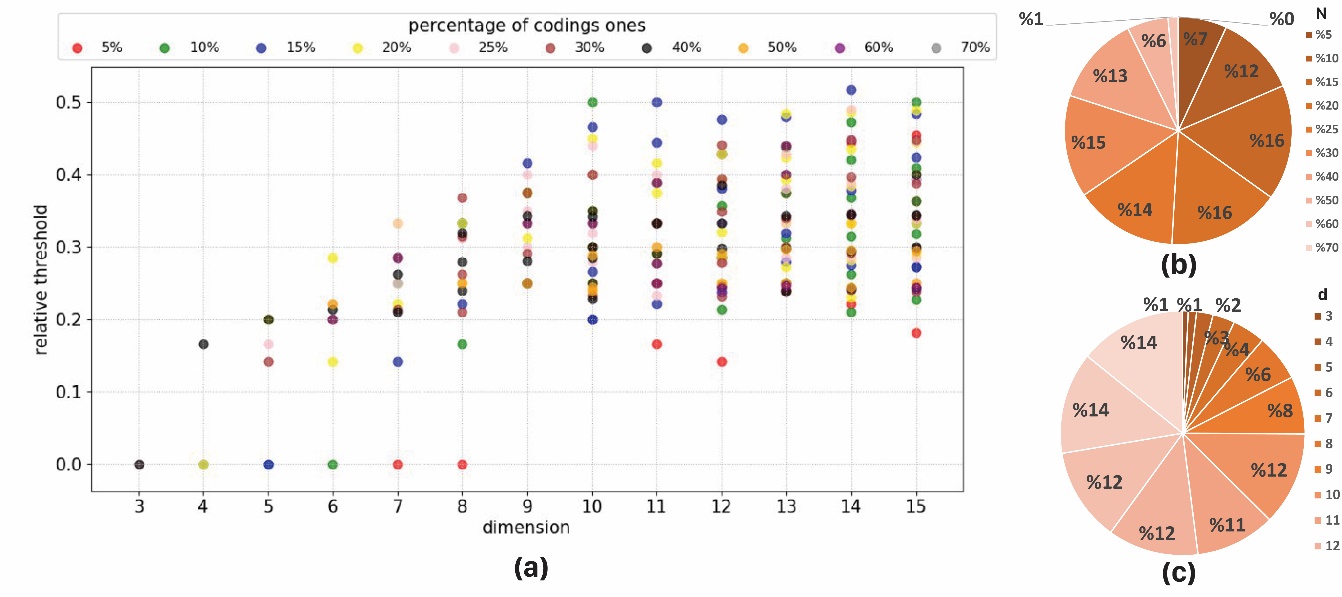


Figure S7 a) Generated zero-score 2D codings for various values of triple d, N and relative threshold, b) zero-score 2D codings distribution according to N, c) zero-score 2D codings distribution according to d

## Concluding remarks

Finally, efficient ratio depends on devices and their applications; thus, choice of suitable parameters for the algorithm depends on existing tradeoffs between length of sequences and required ratio for accurate analysis of device output. in this way, the user according to his/her device requirements and information as shown in Figure S6 and Figure S7, can decide on the value of parameters d, N and E to generate his/her desired code. For example, one criterion for choosing E is system noise amount in device. Considering ε as the least upper bound for system noise in the longest possible peak on output surface, so 2ε becomes a lower bound for E (these boundaries for an informative peak are shown in Figure S2 too); because least difference between two encoded strings, i.e. a single mutation, should be confidently detectable. A lower bound on E restricts choice of size, and target ratio. Of course, for d > 10 and small values of N, the random mode of generating codes will often receive an acceptable score. It might seem that there is no need to use the GAC method, but the point is, in fact, we pay the area-cost of occupying more space on SLM by larger coding instead of the time-cost of the GAC method. However, by doing this, we increase execution time of the cross-correlation due to the more number of data switching on SLM in the case of long sequences, and so, randomized coding sets do not necessarily meet the time limitations.

Concluding the section, Table S*III* summarizes the impact of each parameter, i.e. relative threshold, N, and d, on different metrics evaluating a proper coding set.

Table SIII Effectiveness of optimizing triple parameters (i.e. relative threshold, E, and N) and coding metrics

| Metrics | Relative threshold | N | d |
| --- | --- | --- | --- |
| Overlap noise | Inverse | Small N: Inverse, Large N: direct | Inverse |
| Neighbor noise | Inverse | Small N: Inverse, Large N: direct | Inverse |
| Number of zero-score coding sets | Inverse | Small N: direct, Large N: Inverse | Direct |
| Population time | Direct | Small N: Inverse, Large N: direct | Direct |
| Evolution time | Direct | Small N: Inverse d, Large N: direct | Inverse |

Top of FormBottom of Form
